# Eye-Tracking and Borderline Personality Disorder: A Systematic Review

**DOI:** 10.3390/brainsci16070712

**Published:** 2026-07-01

**Authors:** Marcelo Leiva-Bianchi, Marcelo Nvo-Fernández

**Affiliations:** 1 Laboratory of Methodology, Behavioural Sciences and Neuroscience, Faculty of Psychology, Universidad de Talca, Talca 3460000, Chile; marcleiva@utalca.cl; 2 Psychology Program, Faculty of Social Sciences and Humanities, Universidad Autónoma de Chile, Talca 3460000, Chile

**Keywords:** borderline personality disorder, eye-tracking, pupillometry, attentional bias, emotion dysregulation, saccades, systematic review, PRISMA

## Abstract

**Highlights:**

**What are the main findings?**

- Across the seventeen studies reviewed, BPD patients showed a two-stage attentional pattern: fast initial fixations on the eye region of emotional and neutral faces, with shorter time spent on positive stimuli during prolonged viewing.
- Self-reported impulsivity was high, but performance on standard laboratory inhibition tasks was mostly intact. The deficits that did appear were limited to preparatory oculomotor control, and were larger in patients with comorbid ADHD, psychotic-like symptoms, or under induced anger or anxiety.

**What are the implications of the main findings?**

- Eye-tracking and pupil measures may complement self-report instruments in the clinical assessment of BPD, particularly to distinguish patients in whom the threat-related bias predominates from those with reduced engagement towards rewarding social cues.
- Intranasal oxytocin reduced amygdala-driven vigilance and partly normalised gaze behaviour, which lends preliminary support to combined approaches integrating pharmacological adjuncts with Dialectical Behaviour Therapy.

**Abstract:**

**Background/Objectives**: Borderline personality disorder (BPD) is a severe mental disorder characterised by emotion dysregulation, impulsivity and interpersonal hypersensitivity. Its prevalence ranges from 0.5% to 6.4%. Eye tracking and pupillometry provide objective indices of social attention and inhibitory control, but the BPD literature using these techniques has not been systematically reviewed. The aim of this work was to synthesise the empirical evidence on visuo-attentional and pupillary alterations in BPD. **Methods**: Following the PRISMA 2020 statement, Web of Science, Scopus and PubMed were searched up to 13 March 2026, with no date or language restrictions. Search terms combined borderline personality disorder and eye-tracking constructs. Two reviewers independently screened records with complete inter-rater agreement at the title-and-abstract stage (Cohen’s κ = 1.00); two generative artificial-intelligence assistants (ChatGPT, NotebookLM) were additionally consulted as a non-systematic plausibility check and returned no eligible studies beyond the database search. Risk of bias was appraised with the framework appropriate to each study design (RoB 2 for randomised trials and Newcastle–Ottawa Scale logic for observational studies, with ROBINS-I held in reserve for non-randomised intervention designs). **Results**: Seventeen studies met the inclusion criteria, with sample sizes ranging from 19 to 164 participants and predominantly adult female samples. Designs included antisaccade and oculomotor tasks, free-viewing, dot-probe, affective priming and pharmacological challenge. Four findings recurred across studies. First, patients with BPD showed an early reflexive vigilance to the eye region of emotional and neutral faces, followed by reduced time on positive stimuli during longer presentations. Second, self-reported impulsivity was elevated, but laboratory inhibition was largely preserved; the deficits that did emerge were limited to preparatory control and were greater in patients with comorbid ADHD or under induced negative affect. Third, autonomic dysregulation was indexed by lower heart-rate variability and a larger baseline pupil size; in a single longitudinal study, pupillary reactivity was prospectively associated with subsequent symptom change. Finally, intranasal oxytocin reduced amygdala-driven vigilance. **Conclusions**: Eye-tracking and pupillometric measures appear to capture meaningful aspects of the BPD clinical picture. The two-stage profile of early vigilance followed by reduced sustained engagement is most parsimoniously described as a vigilance–avoidance pattern, which is compatible with, but not uniquely explained by, the hypersensitivity hypothesis of emotion dysregulation. Because thirteen of the seventeen studies recruited women only, these conclusions apply primarily to adult women with BPD. Methodological heterogeneity, the predominance of female samples and the scarcity of longitudinal data justify the need for standardised protocols, transdiagnostic comparisons and the inclusion of male and gender-diverse populations in future research.

## 1. Introduction

Borderline personality disorder (BPD) is a pervasive pattern of instability in interpersonal relationships, self-image and affect, accompanied by marked impulsivity [1]. Three core symptom dimensions have been described. The first is emotion dysregulation, which encompasses impulsive behaviours under stress, and includes self-injury and suicidal ideation. The second is identity disturbance, manifested as an unstable sense of self. The third is interpersonal dysfunction, expressed in unstable relationships and an intense fear of abandonment [2]. The clinical impact of these symptoms is substantial: BPD is considered a severe mental disorder with marked functional impairment [3], with a community prevalence ranging between 0.5% and 3.2% and rates between 2.1% and 6.4% in psychiatric samples [4,5].

Previous meta-analytic evidence indicates that BPD is associated with altered social-affective processing, including heightened threat sensitivity, greater attention to negative stimuli [6] and atypical patterns of gaze during interpersonal interaction [7]. Neuropsychological meta-analyses point to executive dysfunction in domains spanning attention, cognitive flexibility, learning, memory, planning, processing speed and visuospatial functioning [8], although the magnitude and even the presence of these deficits are inconsistent across studies and appear to be substantially attenuated once comorbid conditions, current affective state and medication status are taken into account, so that it remains debated whether executive dysfunction is intrinsic to BPD or largely secondary to these moderators. Neuroimaging work has documented reduced activation in the dorsal anterior cingulate cortex, inferior frontal gyrus and inferior parietal sulcus [9]. A meta-analysis of event-related potentials in BPD has linked these alterations to P300 modulations and to the social-determinant context in which BPD develops [10].

Eye tracking provides objective indices of social attention and inhibitory control [11], and prior research has shown that individuals with BPD exhibit altered processing of emotional faces [12,13], although findings differ as to whether the predominant pattern is one of hypervigilance or of reduced engagement with positive cues. The relationship between BPD and eye-tracking outcomes has nevertheless not been systematically reviewed: a single transdiagnostic systematic review of eye-tracking biomarkers of inattention included only one BPD study [14]. The purpose of the present work is to address this gap by providing a BPD-specific synthesis of the eye-tracking and pupillometric literature, in line with the scope of the Special Issue “Eye-Tracking Monitoring of Neurological and Psychiatric Conditions Across Life Span” in Brain Sciences.

## 2. Materials and Methods

A systematic review was conducted following PRISMA recommendations [15]. Searches were performed in Web of Science, Scopus, and PubMed without language or date restrictions up to 13 March 2026. The search strategy targeted topic, title, abstract, and keywords. It combined terms related to borderline personality disorder and eye-tracking. The strategy included borderline personality disorder, emotionally unstable personality disorder, borderline traits, gaze, visual scanning, fixation, saccades, antisaccades, pupillary responses, oculomotor processes, attentional bias, and scanpath. The strategy was adapted to each database (Appendix A). The databases were searched without language restrictions; all records that ultimately met the eligibility criteria were nevertheless published in English, and no eligible non-English record was identified. Three core bibliographic databases were searched (Web of Science, Scopus, and PubMed). PsycINFO was not searched directly; its omission is acknowledged as a limitation in Section 4.6, although the substantial indexing overlap between PsycINFO and the three databases searched, together with the parallel artificial-intelligence-assisted retrieval, makes it unlikely that a large number of eligible records was missed. A post hoc verification search that additionally included the term “eye movement*” returned no further eligible eye-tracking studies; the records it added corresponded predominantly to eye-movement desensitisation and reprocessing (EMDR) therapy trials, which do not use eye tracking as a measurement technique and were therefore not eligible.

**Figure 1:**
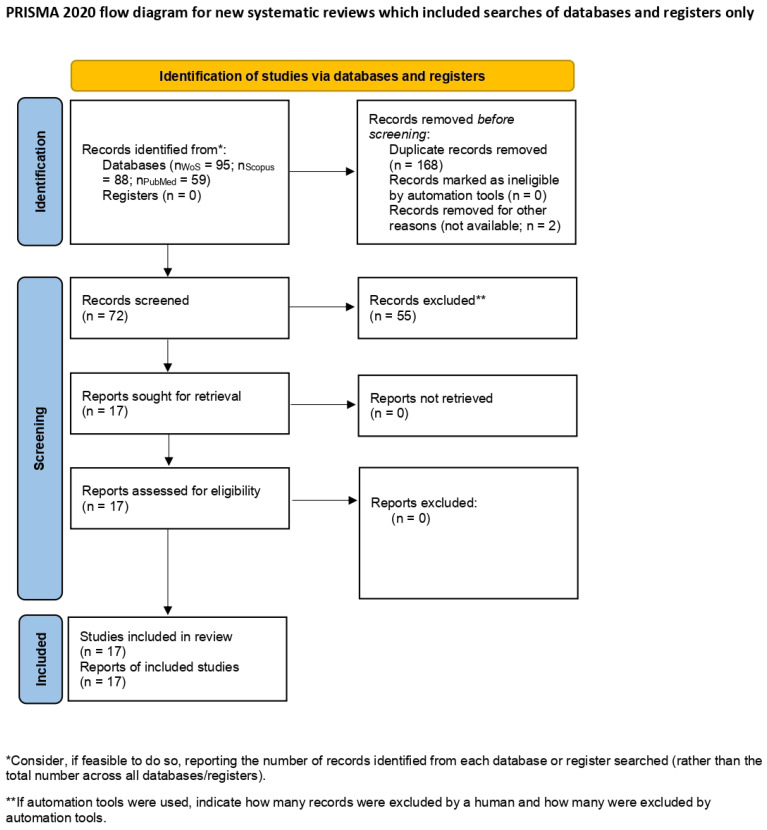
PRISMA flow diagram (Page MJ, et al. BMJ 2021) [15].

Eligible studies were primary empirical investigations on BPD and eye-tracking. Reviews, theoretical papers, and studies focused only on BPD or only on eye tracking were excluded (Figure 1). Records were accessed through institutional subscriptions. Two reviewers (M.L.-B. and M.N.-F.) independently screened titles and abstracts of all 72 deduplicated records (an additional 2 records identified by the database searches were not retrievable and removed before screening; see Figure 1) and reached complete agreement (Cohen’s κ = 1.00) [16]. Complete agreement at this stage is attributable to the narrowness and conjunctive nature of the eligibility rule: a record was retained at the title-and-abstract stage only if it jointly referred to borderline personality disorder (or an explicit synonym) and to an eye-tracking, oculomotor or pupillary measure. Because this conjunction was, in practice, unambiguous from the title and abstract of the deduplicated records, the two reviewers classified every record identically; the agreement therefore reflects the low ambiguity of a small and highly specific record set rather than a claim of perfect reliability that would generalise to broader or fuzzier screening tasks. Residual judgement was deferred to the full-text stage, where eligibility was re-assessed in detail. At the full-text eligibility stage, all seventeen reports retrieved met the inclusion criteria and were assessed independently by both reviewers, who reached complete agreement; no exclusions were applied at this stage and no third adjudicator was required. The seventeen studies were therefore included in the final qualitative synthesis. Risk of bias was appraised with the framework appropriate to each study design: RoB 2 for randomised controlled trials [17,18], ROBINS-I for non-randomised studies of interventions where applicable, and Newcastle–Ottawa Scale (NOS) logic adapted for observational case-control and cohort studies, which constituted the majority of the corpus. In practice, the corpus comprised two randomised controlled trials (appraised with RoB 2) and fifteen observational studies (appraised with NOS logic); no non-randomised study of an intervention met the conditions for ROBINS-I, which was therefore held in reserve but not ultimately applied. The framework applied to each study is reported in the Quality appraisal column of Table 2, and overall quality was summarised on a four-level scale (Low, Moderate, Some concerns, High) to allow comparable narrative synthesis across frameworks. Because the three appraisal tools do not share a common metric, the four-level scale was applied as a harmonised summary judgement derived from the domain-level signals of each tool, according to the following rule. For randomised controlled trials appraised with RoB 2, a study rated “Low” risk across domains was labelled High quality, a rating of “some concerns” was carried over as Some concerns, and “high” risk in one or more domains was labelled Low quality. For observational studies appraised with Newcastle–Ottawa Scale logic, studies satisfying most criteria across the selection, comparability and outcome domains (broadly equivalent to 7–9 of 9 stars) were labelled High quality, those satisfying an intermediate proportion (broadly 4–6 stars) were labelled Moderate, and those satisfying few criteria (broadly ≤3 stars) were labelled Low quality; the intermediate “Some concerns” label was reserved for studies with an otherwise Moderate-to-High profile weakened by a single salient threat to validity (for example, substantial unaddressed missing data). Two reviewers assigned each summary judgement independently and resolved discrepancies by consensus. This harmonisation is interpretive rather than quantitative and is reported only to aid comparison across designs; the underlying tool-specific signals for each study are retained in the Quality appraisal column of Table 2. This systematic review was reported in accordance with the PRISMA 2020 statement [15], and the completed PRISMA 2020 checklist is provided as Supplementary Materials (Table S1). The review was not registered in PROSPERO or any other registry, and no a priori protocol was published; consequently, no registration number is available. This choice is acknowledged as a limitation in Section 4.6, and the eligibility criteria, screening procedure and synthesis plan are reported in full in the present section to support transparency and reproducibility in the absence of a registered protocol.

Data extraction was performed by both reviewers (M.L.-B. and M.N.-F.) using a standardised template in which sample characteristics, recruitment setting, study design, eye-tracking instrument and sampling rate, task or paradigm, primary and secondary outcomes, and quality-related variables were recorded. Each report was extracted independently by one reviewer and verified by the other; discrepancies were resolved by re-reading the source and reaching consensus. No automation tools were used to extract data from the reports included.

Given the methodological heterogeneity of the included studies in terms of paradigms (antisaccade, metronome, free-viewing, dot-probe, affective priming, oxytocin challenge), eye-tracking instruments, sampling rates, areas of interest and reported metrics, no quantitative pooling of effect sizes was performed. The synthesis was therefore narrative and structured around the thematic subsections reported in Section 3, with effect estimates from individual studies (test statistics, *p* values and effect sizes where available) reported verbatim in Table 1 and Table 2 rather than recomputed. Studies were grouped according to outcome family (visuo-attentional, oculomotor inhibition, autonomic, pupillary) and according to whether they involved pharmacological challenge or interpersonal stress induction. Heterogeneity was assessed qualitatively by inspection of paradigm, sample and outcome characteristics rather than statistically.

A formal certainty-of-evidence assessment using GRADE was not performed because the primary outcomes of the corpus were heterogeneous oculomotor and physiological indices rather than effect estimates of comparable interventions amenable to GRADE rating across studies. Study-level methodological quality was instead summarised on the four-level scale reported in the Quality appraisal column of Table 2, with the framework applied to each study (RoB 2 for randomised trials or NOS-adapted logic for observational studies) declared explicitly. Reporting bias at the level of the corpus could not be evaluated formally through funnel-plot asymmetry tests because no quantitative meta-analysis was conducted. The presence of studies reporting null findings on several outcomes (for example, no group differences in saccade main sequence or in temporal prediction accuracy) shows that the published evidence is not exclusively positive, but it does not by itself exclude publication or selective-reporting bias: the included primary studies generally had small samples, were rarely preregistered, and frequently relied on exploratory analyses with multiple outcomes, all of which increase the risk of selectively reported effects. The strength of the evidence underlying the main conclusions of this review should therefore be regarded as low to moderate in formal terms. The recurrent qualitative patterns (early vigilance to the eye region, reduced sustained engagement with positive cues, a self-report–performance dissociation in impulsivity, and autonomic dysregulation) are supported by convergence across independent laboratories and paradigms, which lends them qualitative robustness; however, the magnitude of these effects, their consistency across sexes and age groups, and their diagnostic specificity cannot be established from the present corpus and await confirmation in larger, preregistered, and more demographically representative studies.

As a supplementary, non-systematic plausibility check, and for full transparency, the same eligibility criteria were additionally provided to two generative artificial-intelligence assistants (ChatGPT, OpenAI; and NotebookLM, Google) during the screening period (March 2026). Each tool was prompted, in natural language, to list published empirical studies that jointly investigated borderline personality disorder and eye-tracking, oculomotor or pupillary measures; the records they returned were then checked by the two human reviewers against the same eligibility criteria applied to the database results. These tools returned a small number of candidate records, all of which were either already identified through the database search or, on inspection, ineligible (for example, records that did not use eye-tracking methodology or did not concern borderline personality disorder). No additional eligible study was identified through this procedure. Because large language models do not query bibliographic indices deterministically and do not return reproducible, version-stable result sets, this artificial-intelligence-assisted check is explicitly not considered part of the systematic search strategy, is not represented as an information source in the PRISMA flow diagram, and was used only as an auxiliary cross-check; the systematic identification of studies rests entirely on the three bibliographic databases. The outputs of these tools were not used for screening decisions, data extraction, risk-of-bias appraisal or synthesis, all of which were performed exclusively by the human reviewers.

## 3. Results

Following Brain Sciences reporting recommendations, the results are organised into thematic subsections. The first three subsections describe sampling, study design and outcomes (Table 1). The next two address study quality and ethical considerations (Table 2, with the full per-study appraisal in Supplementary Table S2). The final five synthesise the main substantive findings (Table 2).

### 3.1. Recruitment Settings and Sampling Characteristics

Recruitment and sampling strategies across the corpus are highly specialised, focusing primarily on clinical populations and matched healthy controls. Most studies drew participants from tertiary-care university hospitals and specialised outpatient clinics, including the University Medical Center Freiburg [19], the University of Heidelberg [17,20,21], the Central Institute of Mental Health and Medical Faculty Mannheim of Heidelberg University [22], the University of Rostock [18], San Raffaele Hospital in Milan [23,24,25], the University of Leipzig [26,27], the Queen’s Eye Movement Lab and Kingston Health Sciences Centre [28,29,30], the University of Pittsburgh [31], Radboud University Medical Center [32] and a multi-centre German–Dutch consortium [33]. Several clinical samples were drawn specifically from specialised Dialectical Behaviour Therapy (DBT) outpatient programmes [28,29,30].

A majority of studies sampled female participants exclusively to maintain group homogeneity, citing reported sex differences in BPD symptom presentation [17,18,19,20,21,22,23,26,27,30,31], and adolescent and paediatric studies also focused exclusively on female participants [28,29,30,31]. Only two studies included both male and female participants, with females still predominating [24,25]. Sample sizes per group typically ranged from 14 to 57 participants, with larger samples in fMRI-integrated studies [18] and in the multi-centre diagnostic protocol [33]. Healthy control or non-patient comparison groups were systematically matched for age, intelligence and educational level [17,19,22,27]. Diagnostic confirmation was usually obtained through Structured Clinical Interviews for DSM-IV or DSM-5 (SCID-I, SCID-II or SCID-5-PD), which were also used to screen for comorbidities [19,20,23,25,29,30]. Common exclusion criteria included psychotic disorders, bipolar I disorder and current substance dependence [17,19,22,27]. Many studies required participants to be medication-free or to undergo a wash-out period of at least two weeks before testing [17,18,19,20,21,22], whereas others included medicated patients but documented or controlled for the prescribed psychotropic drug classes [28,29,30].

### 3.2. Experimental Design, Tasks, and Interventions

#### 3.2.1. Oculomotor and Inhibition Tasks

Three task families were used to probe motor synchronisation and executive inhibition. The classical antisaccade task, in which a reflexive prosaccade has to be inhibited and a voluntary saccade made in the opposite direction, was used in three studies [19,29,32]. Two further oculomotor paradigms were used in paediatric BPD samples: the metronome and random tasks, which evaluate temporal motor prediction by asking participants to synchronise eye movements with predictable or unpredictable target pacing [28], and the interleaved pro-/antisaccade task (IPAST), which requires rapid switching between task sets [29]. A competitive mixed-strategy game using saccadic responses (Matching Pennies) was also used to characterise waiting impulsivity in adolescent BPD [30].

#### 3.2.2. Social and Emotional Processing Tasks

Four paradigms were used to test the hypersensitivity hypothesis at distinct levels of processing. Emotion classification paradigms with brief (150 ms) and long (5000 ms) presentations of facial expressions (angry, fearful, happy and neutral) were used to dissociate reflexive from sustained attentional processes [17,20,22]. Free-viewing tasks, in which several facial expressions or complex social scenes are presented simultaneously without explicit task instructions, allowed self-generated attentional deployment to be characterised [18,23,26]. Affective priming paradigms with masked emotional primes followed by neutral target faces were used to assess automatic evaluative processes and gaze orientation [27], and a relational dot-probe task using socio-relational images (positive, negative and erotic) was used to measure initial attentional capture in interpersonal contexts [25].

#### 3.2.3. Contextual and Pharmacological Interventions

Two studies used double-blind placebo-controlled designs to test the effect of intranasal oxytocin (24–26 IU) on social threat hypersensitivity [17,18]. One longitudinal community study used recordings of maternal feedback (criticism, praise and neutral comments) to elicit emotional reactivity and to relate pupillary responses to subsequent BPD symptom development [31].

### 3.3. Primary and Secondary Outcomes, Instruments, and Measures

The reviewed studies adopt a multimodal approach to evaluate the clinical phenotype of BPD. The primary eye-tracking outcomes can be grouped into three families. Temporal indices include first-fixation latency, entry time to the area of interest, saccadic reaction time and peak velocity [17,20,23,26,27,29]. Spatial and preference indices include dwell time, proportion of fixations within the area of interest, number of fixation changes and initial allocation or capture [20,22,23,25,26,33]. Oculomotor error indices include saccade direction errors, anticipatory saccades and fixation breaks [28,29,30,32]. These eye-tracking outcomes are complemented by primary physiological measures: heart-rate variability indices such as RMSSD, HF-HRV and SDNN, as markers of autonomic tone and emotional regulation [23]; mean pupil size and pupil dilation magnitude in response to emotional or cognitive load [28,31]; and electrodermal activity, including skin conductance response latency and level [23]. Secondary behavioural outcomes include accuracy and manual reaction times [17,19,22,25] and subjective ratings of valence, arousal and dominance using affective sliders [23,24,27].

A range of standardised psychometric instruments was used across studies to characterise BPD severity, impulsivity, trauma exposure and affective state. BPD symptom severity was most often measured with the Borderline Symptom List 23 (BSL-23) [17,20,27,28,29,30], the BPD Severity Index (BPDSI) [19] and the Personality Assessment Inventory–Borderline Features Scale (PAI-BOR) [21]. Impulsivity was assessed with the Barratt Impulsivity Scale (BIS-11) [19,28,29,30], the I-7 and the UPPS [19]. Childhood trauma and current affective state were quantified with the Childhood Trauma Questionnaire (CTQ) [21,22,26,27], the Beck Depression Inventory-II and the State–Trait Anxiety Inventory [17,20,24,27], with additional use of the Difficulties in Emotion Regulation Scale [20,22,30] and the Personality Inventory for DSM-5 [23,24].

### 3.4. Methodological Quality and Risk of Bias

#### 3.4.1. Study Design and Selection Bias

The reviewed corpus is dominated by case-control experimental designs [17,18,19,20,21,22,23,24,25,26], with two randomised placebo-controlled studies of intranasal oxytocin [17,18] and one prospective community cohort [31]. Patients with BPD and healthy controls or non-patient comparison groups were consistently matched on age, intelligence and educational level [17,19,20,21,22,24,26,27,28,29]. A clear gender-based selection bias is nevertheless present: thirteen of the seventeen included studies sampled female participants exclusively to maximise group homogeneity, motivated by reported sex differences in BPD symptom expression and brain circuitry [17,18,19,20,21,22,24,26,27,28,29,30,31]. Diagnostic ascertainment was generally rigorous, with structured interviews such as the SCID-I, SCID-II or SCID-5-PD applied across most studies [17,18,19,20,21,22,24,25,26,30,31].

#### 3.4.2. Confounding Factors and Comorbidity Management

Psychiatric comorbidity is a primary source of confounding in this literature. Most studies document elevated rates of comorbid depression, anxiety, post-traumatic stress disorder and eating disorders [17,20,21,23,24,25,26,27,29,31,32]. Approaches to handling these confounders varied: some studies controlled for symptom dimensions such as depressive or anxious symptoms as covariates [17,23,27], while others addressed comorbidity by stratifying the BPD sample, for example into participants with and without comorbid ADHD [28,29,30] or with and without psychotic-like symptoms [32]. Medication exposure was managed through two main strategies. Some studies recruited unmedicated patients [17,26,27], while others included medicated patients but documented prescriptions and conducted sensitivity analyses [20,22,23,24,25,29,31]. Only a minority of studies imposed a full two-week washout period for psychotropic medication on all participants [19,26,27].

#### 3.4.3. Outcome Measurement Quality and Missing Data

Outcome measurement was characterised by high technical rigour. Sampling rates ranged from 60 Hz on consumer-grade or MRI-compatible video-based systems [17,18,20,21,23,24,25,31], through 250 Hz on remote video-based systems [26,27], to 500 Hz on research-grade systems [28,30]. One study recorded eye position via electro-oculography rather than video-based tracking [32], and the remaining studies did not explicitly report the sampling rate in the main text [19,22,29,33]. Calibration accuracy was tightly controlled, with most studies requiring deviations of less than 0.5° to 1.5° from target [17,21,22,23,24,25,26,27]. Substantial participant exclusion was nevertheless reported across studies as a result of recording artifacts, calibration failure or technical malfunction, with exclusion rates of 10–25% of the initial sample in several reports [17,21,23,25,26,27,28]. The risk of selective reporting was mitigated by the use of established a priori hypotheses and standardised experimental paradigms such as IPAST, free-viewing and emotion classification [17,20,21,22,24,25,26].

### 3.5. Ethical Considerations and Transparency

#### 3.5.1. Ethics Approval and Informed Consent

Ethical standards are documented across the corpus. All protocols received approval from local institutional review boards or university ethics committees, including those of Heidelberg, Freiburg, Milan, Queen’s University and Leipzig, and written informed consent was obtained from all adult participants. Studies involving paediatric or adolescent patients aged 11–18 years followed enhanced ethical procedures, combining written parental consent with oral participant assent for younger participants and written consent for those at least 17 years of age [28,29,31].

#### 3.5.2. Risk–Benefit Balance and Privacy

In pharmacological [17,18] and stress-induction [31] designs, participants were monitored for adverse effects, and no serious adverse events were reported. Participant data were handled using anonymised identifiers across all studies, and financial reimbursement or hourly compensation was standard practice at European and North American sites [17,21,22,23,25,29].

#### 3.5.3. Ethical Transparency and Data Availability

Ethical transparency was generally adequate, with explicit conflict-of-interest declarations in several reports [17,24,25,27,29,31] and explicit data availability statements in more recent studies, typically indicating that datasets were available from the corresponding author upon reasonable request [21,22,23,25,26,31]. None of the included studies reported a formal preregistration of the experimental protocol, although one made the materials and the dataset publicly available through the Open Science Framework [22]. Use of generative artificial intelligence in the creation or analysis of the data was not documented in any of the included reports.

### 3.6. Reflexive Visual Attention and Social Threat Sensitivity

Eye-tracking indices converge on early reflexive sensitivity to social cues in BPD [17,19,20]. Compared with healthy controls, patients with BPD orient faster and more frequently toward the eye region of facial stimuli, and this hypervigilance is observed for emotional and neutral expressions alike [19,20]. Some studies report a more specific anger-related bias, with shorter saccadic latencies toward angry eyes, while others describe a generalised vigilance to the eye region irrespective of valence, which is consistent with the eyes acting as the most salient social cue [20]. These two accounts are not equivalent: a general attentional preference for the eye region would be expected to appear across all expressions, including neutral and positive faces, whereas a threat-specific bias would be confined to angry or fearful stimuli. The reviewed evidence is mixed on this point, with some studies finding faster orienting toward eyes regardless of valence and others finding a sharper effect for angry eyes; the present corpus does not allow these alternatives to be decided conclusively, and both a domain-general salience account and a threat-specific account remain tenable. At the neural level, faster initial saccades toward angry eyes are accompanied by greater right posterior amygdala activation, and the latency of the initial saccade toward threatening eyes correlates with self-reported aggressiveness [17].

### 3.7. Sustained Visual Exploration and Attentional Avoidance

Beyond initial orienting, the reviewed studies converge on reduced visual exploration and attentional avoidance during later stages of processing [17,18,21,22]. Dwell time on happy faces is shorter in BPD than in healthy controls, indicating a diminished positivity bias and a poor maintenance of attention on social safety cues [26]. In relational dot-probe and free-viewing paradigms, patients show rapid early orienting toward negative or socio-relational stimuli followed by marked attentional avoidance at later stages, a vigilance–avoidance pattern that is not observed for non-relational material [18]. During prolonged viewing of complex socio-emotional scenes, fixation counts are lower and the time spent within relevant social areas of interest is reduced relative to controls [22,23]. A complementary finding under masked priming, where prime awareness can be ruled out, is that patients with BPD rate neutral target faces as more negative than non-patients, suggesting a pervasive negative interpretation bias that operates independently of conscious gaze deployment [24].

### 3.8. Behavioural Inhibition and Preparatory Control

A recurrent finding in this corpus is the discrepancy between elevated trait impulsivity on self-report measures and largely intact performance on standard inhibition paradigms. Patients with BPD score high on instruments such as the Barratt Impulsivity Scale, the I-7 and the UPPS, but their performance on Stroop, Stop-Signal and antisaccade tasks under emotionally neutral conditions does not differ from that of healthy controls [19,28,29]. The deficits that do emerge involve preparatory control rather than motor execution: delayed acquisition of the initial visual fixation, more frequent fixation breaks during preparatory intervals, and greater variability in saccadic reaction time and peak velocity [29]. Higher rates of anticipatory saccades and direction errors appear when comorbid attention-deficit/hyperactivity disorder is present [28,29,30] and a similar pattern of impaired response inhibition is observed in patients with psychotic-like symptoms [32]. Inhibition errors also increase under induced anxiety or anger states, an effect documented in BPD but not in healthy controls [25].

### 3.9. Physiological and Pupillary Markers

Autonomic dysregulation and elevated sympathetic tone characterise the BPD phenotype across several studies in this corpus [23,28]. Patients with BPD show reduced heart-rate variability indexed by RMSSD and HF-HRV [23], a pattern consistent with meta-analytic evidence of reduced resting vagal tone in BPD [34]. Paediatric patients with BPD exhibit larger baseline pupil sizes than age-matched controls regardless of task demands, suggesting persistent sympathetic hyperarousal [28]. This interpretation should be made with caution: baseline and task-evoked pupil size reflects the joint action of sympathetic and parasympathetic pathways and is additionally shaped by luminance, cognitive load, arousal and locus-coeruleus–noradrenergic activity, so that a larger pupil cannot be read as a pure index of sympathetic tone. The convergence of larger pupil size with independently measured reductions in heart-rate variability nonetheless makes a contribution of altered autonomic balance plausible. In a longitudinal community sample of adolescent girls, stronger pupillary responses to maternal criticism prospectively predicted greater BPD symptom severity over an eighteen-month follow-up, providing developmental support for the role of physiological reactivity in the emergence of BPD features [31].

### 3.10. Pharmacological and Contextual Modulation

Two double-blind placebo-controlled studies in adult women with BPD showed that a single intranasal dose of oxytocin (24–26 IU) reduces amygdala and insula reactivity to social stimuli and attenuates reflexive vigilance toward angry eyes, bringing gaze and neural responses closer to those of healthy controls [17,18]. The effects of contextual variables also matter: behavioural inhibition errors in BPD increase as a function of current anxiety or anger intensity, an interaction that is not present in healthy controls and that suggests state-dependent modulation of executive control rather than a fixed cognitive deficit [25].

**Table 1:** Sample characteristics, study designs, and outcomes.

Study	Recruitment Setting	Sample Analysed	Design	Task/Intervention/Exposure	Outcomes	Instruments or Measures
Bertsch et al., 2013 [17]	University psychiatric setting and community. Germany (Heidelberg)	Adult women (Mage = 24.4; SD = 4.7) with BPD (*n* = 38; Oxytocin Group *n* = 19; Placebo Group *n* = 19) vs. healthy controls (*n* = 41; Oxytocin Group *n* = 21; placebo Group *n* = 20).	Randomised, placebo-controlled and double-blind	Single intranasal dose 26 IU oxytocin vs. placebo. Emotion classification task with angry, fearful, happy faces. Recorded with eye tracking and concurrent fMRI.	Latency and proportion of initial fixation changes; amygdala BOLD response; manual response latency; classification accuracy.	MRI-compatible eye-tracker (video-based). fMRI (3T scanner). Diagnostic interviews (IPDE; SCID). BSL-23. Beck Depression Inventory
Bertsch et al., 2017 [20]	University psychiatric setting and community. Germany (Heidelberg)	Adult women with BPD (Mage = 25.3; SD = 5.6; *n* = 20) vs. healthy volunteers (Mage = 25; SD = 3.6; *n* = 24).	Case-control	Emotion classification task with eye-tracking. Faces (angry, fearful, happy, neutral). Presentation times 150 ms vs. 5000 ms. Manipulated initial fixation (eyes vs. mouth).	Proportion and latency of initial saccades; fixation duration on facial features; emotion recognition accuracy. Response latency. Error types.	Arrington ViewPoint eye-tracker. SCID-I. IPDE. Aggression Questionnaire. BSL-23. BDI-II. STAI. STAXI. DERS. MDBF
Bortolla et al., 2019 [23]	Mental health centre and community. Italy (Milan)	Adults with BPD (Mage = 28.4; SD = 7.4; *n* female = 11; *n* male = 3) vs. healthy controls (Mage = 27.6; SD = 5.9; *n* female = 10; *n* male = 4).	Case-control	Viewing 48 socio-emotional pictures (Nencki Affective Picture System). Two exposure durations (5 s vs. 15 s). Multimodal assessment: eye tracking, electrodermal activity, ECG, self-report ratings.	SCR latency; eye-tracking indices (prop gaze, fixation duration, mean time). RMSSD modulation; self-reported valence; arousal; dominance.	SCID-II. PANAS. DERS. PID-5. Eye Tribe eye-tracker. BITalino physiological recording
Bortolla et al., 2020 [24]	Mental health centre and community. Italy (Milan)	Adult women with BPD (Mage = 22.9; SD = 5.9; *n* = 20) vs. healthy controls (Mage = 23.6; SD = 5.5; *n* = 20).	Case-control	Viewing 54 socio-emotional pictures (positive. negative. neutral) from Nencki Affective Picture System. Three exposure durations: 500 ms. 3 s. 18 s. Eye tracking and affective ratings collected.	Valence ratings. Eye-tracking indices: proportion of fixations, proportion of time in social AoIs, latency to first fixation. Arousal and dominance ratings; associations with psychopathology traits.	Eye Tribe eye-tracker. Affective Slider scales. PANAS. DERS. PID-5. AAQ-II
Bortolla et al., 2023 [25]	Mental health centre and community. Italy (Milan)	Adults with BPD (M age = 23.2, SD = 3.7; *n* females = 27; *n* males = 4) vs. healthy controls (M age = 22.9, SD = 1.9; *n* females = 27; *n* males = 4).	Case-control	Modified relational dot-probe task using interpersonal pictures (positive. negative. erotic) vs. neutral stimuli. Eye tracking recorded.	Bias score. Reaction time latency. Eye-tracking indices (initial allocation. Latency. time in. duration). Correct responses. PANAS positive/negative affect modulation.	Eye Tribe eye-tracker. PANAS. SCID-5-PD. PID-5. DERS
Calancie et al., 2023 [28]	Child mental health centre and community. Canada (Ontario)	Adolescent females (ADHD/BPD *n* = 22; BPD *n* = 23; Controls *n* = 35).	Case-control	Eye-tracking metronome task (predictable timing). random timing task, saccade synchronisation paradigm.	Percentage predictive saccades. Saccade reaction time. blink rate. Pupil size; blink duration. Blink probability timing. Saccade amplitude and velocity.	SR Research EyeLink 1000 Plus eye-tracker. Barratt Impulsivity Scale. Borderline Symptom List-23. SBQ
Calancie et al., 2024 [29]	Child mental health centre and community. Canada (Ontario)	Adolescent females (BPD *n* = 24; ADHD/BPD *n* = 25; controls *n* = 53).	Case-control	Interleaved pro-/antisaccade task (IPAST) assessing preparatory fixation control and response inhibition.	Fixation acquisition. Fixation breaks. Anticipatory saccades. Express direction errors. Antisaccade error rate. Saccade reaction time variability. Peak velocity variability. Corrective saccade timing.	Video-based eye-tracking. Barratt Impulsivity Scale
Grootens et al., 2008 [32]	Mental health centre and community. Netherlands (Nijmegen)	Adults with BPD psychotic-like symptoms (Mage = 29.5; SD = 6.3; n female = 19; *n* male = 1) vs. BPD patients without psychotic-like symptoms (Mage = 27.6; SD = 6.1; n female = 10; *n* male = 2) vs. schizophrenia patients (Mage = 27; SD = 9.1; *n* female = 4; *n* male = 17) vs. healthy controls (Mage = 25.7; SD = 5.5; *n* female = 11; *n* male = 14).	Case-control	Antisaccade and prosaccade eye-movement task using LED targets.	Percentage inhibition errors (antisaccade task). Percentage anticipatory errors. Correct responses. Response latencies.	Electro-oculography (EOG). SCID diagnostic interviews
Jacob et al., 2010 [19]	Mental health centre and community. Germany (Freiburg)	Adult women with BPD (Mage = 29; SD = 5.5; *n* = 15) vs. healthy controls (Mage = 29; SD = 5.5; *n* = 15).	Case-control	Self-report impulsivity scales. Stroop task. Stop signal task. Antisaccade task.	Self-report impulsivity scores. Behavioural inhibition performance indices. Correlations with emotional state. Relations among impulsivity measures.	BIS. Eysenck I-7. UPPS. Stroop interference effect. Stop signal task (% failed inhibitions). Antisaccade error rate
Kaiser et al., 2019 [33]	Multi-centre research collaboration. Germany (Freiburg, Lübeck, Trier) and the Netherlands (Maastricht, Amsterdam)	Adult female BPD patients. Cluster-C clinical controls. Non-patient controls. Exact Ns not visible in opened excerpts.	Case-control	Eye tracking during recognition of ambiguous facial emotional blends.	Fixation duration on eye region. Emotion recognition accuracy.	Eye-tracking paradigm with ambiguous facial expressions
Lischke et al., 2017 [18]	University psychiatric setting and community. Germany (Rostock)	Adult women (Mage = 25.4; SD = 4.6) with BPD (*n* = 47; Oxytocin Group *n* = 23; Placebo Group *n* = 24) vs. healthy controls (*n* = 46; Oxytocin Group *n* = 22; placebo Group *n* = 24).	Randomised, placebo-controlled and double-blind	Intranasal oxytocin (24 IU) vs. placebo. fMRI and eye tracking during viewing of emotional and neutral scenes.	Amygdala and insula activation during scene processing. Functional connectivity. Coupling between amygdala activity and gaze behaviour. Task performance.	fMRI (SPM8). Eye-tracking (relative fixation indices). Behavioural response accuracy/latency
Niedtfeld et al., 2020 [22]	Mental health centre and community. Germany (Mannheim)	Adult women with BPD (Mage = 29.8; SD = 6.8; *n* = 36) vs. healthy controls (Mage = 29.9; SD = 9.5; *n* = 30).	Case-control	Incidental encoding and recognition of positive vs. negative person-related information, with eye tracking during retrieval.	Social cognition. Memory bias. Eye-movement correlates of retrieval; retrieval accuracy for negative vs. positive person attributes.	Response latency. Fixation of spatial locations associated with negative vs. positive information
Parr et al., 2022 [30]	Child and youth mental health centre and community. Canada (Ontario, Kingston)	Adolescent females with BPD (Mage = 16.4; SD = 1.3; range = 14–18; *n* = 27) vs. age-matched female controls (Mage = 15.8; SD = 1.6; range = 14–18; *n* = 27).	Case-control	Colour-based Matching Pennies mixed-strategy game vs. dynamic computer opponent. Eye tracking during 600 trials in 4 runs. Choices indicated by saccadic eye movements.	Saccadic reaction time (median; CV). Percentage of anticipatory trials. Percentage of non-response trials. Probability of win-stay/lose-shift in spatial and colour domains. Choice entropy. Reward rate.	SR Research EyeLink 1000 eye-tracker (monocular, 500 Hz). SCID-5-PD. Barratt Impulsivity Scale (BIS-11). Borderline Symptom List (BSL-23). Difficulties in Emotion Regulation Scale (DERS)
Scott et al., 2017 [31]	Community longitudinal cohort study. USA (Pittsburgh)	Fifty-seven 16-year-old girls.	Longitudinal	Listening to audio clips of own mother providing criticism, praise, or neutral comments while pupillary response recorded.	Pupillary dilation during maternal criticism and praise. Self-reported affective response. Longitudinal change in BPD symptoms.	Pupillometry. Borderline symptom scale (dimensional). Self-report affect ratings
Seitz et al., 2021 [21]	University psychiatric setting and community. Germany (Heidelberg)	Adult female with BPD (Mage = 28.6; SD = 7.5; *n* = 46) vs. healthy controls (Mage = 26.4; SD = 5.5; *n* = 25).	Case-control	Emotion classification paradigm with eye-tracking. Angry, fearful, happy, neutral faces. Brief (150 ms) vs. long (5000 ms) exposure.	Misclassification rates (anger bias). Initial saccade proportion and latency. Fixation duration on eye vs. mouth regions. Association of attention bias indices with CTQ.	Arrington ViewPoint eye-tracker. CTQ. BSL-23. BDI-II. STAI. DERS. Raven matrices
Wenk et al., 2025 [27]	University psychiatric setting and community. Germany (Leipzig)	Adults with BPD (Mage = 27.8; SD = 6.1; *n* female = 26; *n* male = 5) vs. healthy controls (Mage = 26.7; SD = 5.2; *n* female = 26; *n* male = 5).	Case-control	Masked affective priming paradigm with emotional faces (50 ms). Eye tracking during evaluation of neutral target faces.	Probability of first fixation. Early dwell time (first 500 ms). Overall dwell time. Evaluative ratings of neutral faces.	SMI RED250 eye-tracker. BDI-II. STAI-T. CTQ. BSL-23. TMT-B
Wenk et al., 2024 [26]	University psychiatric setting and community. Germany (Leipzig)	Adults with BPD (Mage = 27.8; SD = 6.4; *n* female = 36; *n* male = 7) vs. healthy controls (Mage = 26.9; SD = 5.9; *n* female = 36; *n* male = 7).	Case-control	Multiple-stimulus free-viewing eye-tracking task, simultaneous happy, angry, sad, neutral faces.	Dwell time (late attention allocation). Entry time (initial orienting). Associations between dwell time and clinical variables.	SMI RED250 eye-tracker. BSL-23. BDI-II. STAI-T/S. TAS-20. CTQ. TMT-B

M = mean; SD = standard deviation; *n* = sample size. See Appendix A for the full list of acronyms used throughout the manuscript.

**Table 2:** Summary of methodological quality and key findings of the included studies. A compact summary is presented here; the full per-study appraisal (domain-by-domain risk-of-bias notes and detailed ethical-transparency assessment) is provided in Supplementary Table S2.

Study	Framework & Overall Quality	Key Risk-of-Bias/Quality Concerns	Funding/Conflicts	Main Findings and Conclusion
Bertsch et al., 2013 [17]	RoB 2; Some concerns	Small sample. Female only. Behavioural data exclusions. No clinical control group.	German Federal Ministry for Education and Research grant. No financial conflicts reported.	BPD placebo group showed faster fixation changes and increased amygdala activation to angry faces. Oxytocin reduced fixation changes toward angry eyes and reduced right posterior amygdala activation. Conclusion: Oxytocin reduces social threat hypersensitivity and amygdala hyperreactivity in female BPD patients.
Bertsch et al., 2017 [20]	NOS logic; Moderate	Small sample. Female only. Comorbidities. Subsample for saccade latency analyses.	German Research Foundation. European Research Council. No conflicts declared.	BPD patients had slower overall responses (F = 5.79, *p* = 0.012). Misclassified faces as angry more often in brief condition (interaction F = 3.98, *p* = 0.012). Conclusion: BPD shows specific early bias toward interpersonal threat cues and deficits in detailed processing linked to aggressiveness.
Bortolla et al., 2019 [23]	NOS logic; Moderate	Small sample. Medication use allowed. Missing EDA data. No clinical control group.	NR	BPD showed faster SCR latency (F = 4.39, *p* < 0.05, η^2^ = 0.17). Reduced visual exploration of socio-emotional cues (prop gaze F = 5.73, *p* < 0.05; fixation duration F = 7.64, *p* < 0.05). Conclusion: Results support hypersensitivity and slow return to baseline but not hyperreactivity hypotheses.
Bortolla et al., 2020 [24]	NOS logic; Moderate	Female-only sample. High comorbidity and medication use. No clinical control group.	No specific funding. No competing interests declared.	BPD rated socio-emotional stimuli more negatively (F = 5.04. *p* = 0.03). Conclusion: Emotional dysregulation in BPD reflects negative appraisal bias and reduced social information processing rather than hyperreactivity.
Bortolla et al., 2023 [25]	NOS logic; Moderate	Medication use common. Comorbidity present. No clinical control group.	No conflicts declared. Funding NR.	Higher bias score for erotic stimuli and lower bias score for negative stimuli in BPD (group × condition F = 3.25, *p* = 0.05). Reduced latency to erotic and negative stimuli indicating hypervigilance. Conclusion: BPD shows hypervigilance to relational stimuli with content-specific later attentional patterns and modulation by baseline negative affect.
Calancie et al., 2023 [28]	NOS logic; Moderate	Medication use allowed. Only females. Comorbidities present.	NR	No group differences in temporal prediction during predictable condition. ADHD/BPD showed more anticipatory saccades in random condition (χ^2^ = 11.13, *p* = 0.004). Conclusion: Temporal motor prediction intact in BPD. Response inhibition deficits mainly linked to ADHD comorbidity. Increased sympathetic arousal in BPD.
Calancie et al., 2024 [29]	NOS logic; Moderate	Female-only adolescent sample. comorbidities allowed. Medication status not fully controlled.	Canadian Institutes of Health Research; SEAMO AFP Innovation Fund. No conflicts declared.	Reduced fixation acquisition in BPD and ADHD/BPD vs. controls (χ^2^ = 13.81, *p* = 0.001). Increased fixation breaks (χ^2^ = 22.65, *p* ≈ 1.2 × 10^−5^). Conclusion: BPD involves impaired preparatory oculomotor control; additional response-inhibition deficits linked to ADHD comorbidity.
Grootens et al., 2008 [32]	NOS logic; Moderate	Medication use allowed; unequal sex distribution; heterogeneous clinical sample.	NR	Significant group effect for inhibition errors (F = 10.3, *p* < 0.001). Schizophrenia > BPD > controls. **Conclusion:** Inhibition deficits characterise a subgroup of BPD patients with psychotic-like symptoms and resemble schizophrenia patterns.
Jacob et al., 2010 [19]	NOS logic; Moderate (fair)	Small sample. Female-only sample. Possible residual confounding from lifetime comorbidity.	NR	Higher impulsivity in BPD on most self-report scales (e.g., BIS behavioural mean 2.5 vs. 1.9, *p* < 0.001). No significant group differences in stop signal or antisaccade tasks. **Conclusion:** BPD shows elevated self-reported impulsivity but not clear deficits in laboratory behavioural inhibition. Emotional state may modulate performance.
Kaiser et al., 2019 [33]	NOS logic; Unclear (likely moderate–low; details missing)	High uncertainty due to missing methodological details.	NR	BPD patients fixated eyes longer than non-patients for angry/happy, sad/happy, fearful/sad blends. Effect mainly driven by BPD with PTSD. **Conclusion:** Attention bias toward eyes in ambiguous emotions in BPD may be trauma-related rather than due to emotion recognition deficits.
Lischke et al., 2017 [18]	RoB 2; Moderate-to-Good	Female-only sample. Single-dose design. Behavioural ceiling effects.	Funded by German Federal Ministry of Education and Research. European Research Council. German Research Foundation. No conflicts declared.	After placebo: BPD showed increased amygdala (Z = 3.99 PFWE = 0.006) and insula activity vs. HC. Oxytocin decreased amygdala/insula reactivity in BPD but increased it in HC (interaction Z ≈ 3.67–3.74 PFWE ≈ 0.013–0.016). **Conclusion:** Oxytocin normalises hyperreactive paralimbic responses and abnormal neural–gaze coupling in BPD during scene processing.
Niedtfeld et al., 2020 [22]	NOS logic; Moderate (fair–good)	NR	German Research Foundation grants.	Generalised linear mixed-effects models. Linear mixed models. **Conclusion:** Enhanced retrieval of negative person information may reinforce dysfunctional schemas and interpersonal distrust in BPD.
Parr et al., 2022 [30]	NOS logic; Moderate (fair–good)	Female-only adolescent sample. Medication regimen not interrupted. Single-site recruitment. Comorbid ADHD common in BPD subgroup.	No specific funding statement reported. Authors declared no conflicts of interest.	BPD adolescents showed greater percentage of anticipatory saccadic decisions vs. controls (M = 6.71% vs. 3.32%, β = −0.92, t = −3.73, *p* < 0.001 Bonferroni-corrected). Increased coefficient of variation in saccadic reaction time (M = 32.88% vs. 29.72%, *p* = 0.05 uncorrected). **Conclusion:** Paediatric BPD is characterised by waiting impulsivity (anticipatory saccades) and elevated saccadic response variability during competitive decision-making, with impulsivity and emotional dysregulation contributing to choice variability.
Scott et al., 2017 [31]	NOS logic; Moderate (fair)	Small sample. Female-only. No diagnostic interview confirmation.	NIMH grants (K01 MH086713; R01 MH101088; R01 MH056630; K01 MH101289; K01 MH086811; F32 MH102895). NIDA (R01 DA012237). Office of Juvenile Justice and Delinquency Prevention (2013-JF-FX-0058). FISA Foundation. Falk Fund. No conflicts declared.	Greater pupillary response to criticism predicted increases in BPD symptoms over time. Greater pupillary and positive affective response to praise associated with higher baseline symptoms but faster symptom decline (context-dependent effects). **Conclusion:** Physiological reactivity to interpersonal feedback may function as both risk and protective factor in BPD symptom development depending on context.
Seitz et al., 2021 [21]	NOS logic; Moderate (fair–good)	Female-only. Comorbidity high. No clinical control group. Subsample analyses for saccade latency.	German Research Foundation grants.	No overall group difference in emotion recognition accuracy (~93%). BPD showed more initial saccades in brief condition (F = 6.62 *p* = 0.012 η^2^ = 0.09). **Conclusion:** Findings suggest generalised visual hypervigilance to social cues and ACE-related anger bias in BPD.
Wenk et al., 2025 [27]	NOS logic; Moderate (fair–good)	Comorbidities allowed. Medication heterogeneity. Female-predominant sample.	No external funding (Open Access funding via Projekt DEAL). No conflicts declared.	No group differences in gaze parameters. Significant negative evaluation bias in BPD (group effect F(1,59) = 4.98 *p* = 0.029 ηp^2^ = 0.08). **Conclusion:** BPD characterised by general negative interpretation bias but not altered automatic gaze orienting to masked emotional stimuli.
Wenk et al., 2024 [26]	NOS logic; Moderate (fair–good)	High psychiatric comorbidity. Medication heterogeneity. Free-viewing paradigm limits avoidance interpretation.	No external funding.	Shorter dwell time on happy faces in BPD (t(84) = 2.66 *p* = 0.005 d = 0.57). No group differences in dwell time for angry. **Conclusion:** BPD characterised by reduced sustained attention to positive facial expressions. No evidence for early threat vigilance.

NR = not reported. Framework = risk-of-bias appraisal tool applied to each study (RoB 2 for randomised controlled trials; Newcastle–Ottawa Scale logic for observational case-control and cohort studies); overall quality is the harmonised four-level judgment (Low, Moderate, Some concerns, High) defined in Section 2. The full per-study appraisal, including domain-by-domain risk-of-bias notes and the detailed ethical-transparency assessment, is provided in Supplementary Table S2. See Appendix A for the full list of acronyms used throughout the manuscript.

## 4. Discussion

This systematic review synthesised seventeen empirical studies examining eye-tracking, pupillometry and complementary physiological markers in borderline personality disorder [17,18,19,20,21,22,23,24,25,26,27,28,29,30,31,32,33]. The included paradigms were heterogeneous and ranged from antisaccade and oculomotor tasks to emotion classification, free-viewing, dot-probe, affective priming and pharmacological challenge. Despite this variability, several findings recurred across studies. The discussion that follows is organised around four of these findings, situates them in relation to previous meta-analytic and neurobiological work, considers their clinical and mechanistic implications, and ends with the methodological limitations of the available evidence.

### 4.1. Visuo-Attentional Alterations: Early Vigilance and Reduced Engagement

The reviewed eye-tracking studies show a two-stage attentional pattern in BPD. At early stages of processing, patients showed faster and more frequent reflexive fixations on the eye region of emotional and even neutral faces, with shorter saccadic latencies towards angry eyes and elevated initial-saccade proportions in brief presentation conditions [17,20,21]. This early vigilance was greater in patients with comorbid post-traumatic stress disorder [24], which suggests that trauma history contributes to the attentional bias rather than being merely incidental to it. At later stages, the pattern reversed: dwell time on happy faces was reduced, fixation counts during prolonged viewing of socio-emotional scenes were lower, and a marked attentional avoidance of negative or relational stimuli was observed in dot-probe and free-viewing paradigms [18,21,22,29]. When this is combined with the negative interpretation bias for ambiguous and neutral material observed under masked priming [27], the resulting picture is compatible with the hypersensitivity hypothesis of BPD emotion dysregulation [2,18] and with meta-analytic evidence that threat-related attentional biases are reliably observed across emotional disorders [35]. It is important to note, however, that the two-stage profile is open to more than one theoretical reading. The hypersensitivity account attributes both the early vigilance and the later reduction in engagement to heightened sensitivity to social-emotional cues, but the same data are at least as well described by a vigilance–avoidance model, in which an initial automatic orientation toward salient or threatening cues is followed by motivated disengagement that down-regulates aversive arousal. The reduced sustained exploration of socio-emotional scenes and the shortened dwell time on positive faces, in particular, may reflect avoidance or strategic regulation rather than a failure of sensitivity. A third possibility is that reduced engagement with positive cues reflects diminished reward salience rather than threat-driven avoidance. The present evidence does not adjudicate decisively among these accounts, and we therefore present the vigilance–avoidance pattern as the most descriptively neutral summary, with hypersensitivity, avoidance-based regulation and reduced positivity each remaining viable mechanistic interpretations. What the data do consistently indicate is that the dysregulation does not appear to reflect indiscriminate hyperreactivity, but rather an altered, time-dependent allocation of attention to socially relevant cues.

### 4.2. Behavioural Inhibition: A Discrepancy Between Self-Report and Performance

A second consistent finding concerned the discrepancy between elevated trait impulsivity on self-report measures (BIS, UPPS, I-7) and largely preserved performance on standard objective inhibition paradigms (Stroop, Stop-Signal, Antisaccade) under neutral conditions [19,28,29]. Where objective deficits did emerge, they clustered in preparatory control rather than in motor execution. Patients with BPD showed delayed initial fixation acquisition, more fixation breaks during preparatory intervals, greater variability in saccade reaction time and peak velocity, and a higher rate of anticipatory saccades and direction errors [29,30]. These deficits were notably larger in patients with comorbid ADHD [28,29,30] or psychotic-like symptoms [32], and inhibition errors increased under induced anxiety or anger states only in the BPD group [25]. The convergence on increased anticipatory saccades across an antisaccade task in paediatric BPD [29] and a competitive mixed-strategy game in female adolescents with BPD [30] suggests that waiting impulsivity is detectable across paradigms, although the effect was driven mainly by patients with comorbid ADHD when the two cohorts were analysed separately [29]. This pattern qualifies the conclusions of Ruocco’s [8] earlier neuropsychological meta-analysis. Frank executive dysfunction may be more attributable to comorbid pathology and to the emotional load present at the moment of testing than to BPD itself, which has clear implications for differential diagnosis. The dissociation between markedly elevated self-reported impulsivity and largely preserved laboratory inhibition is itself one of the more theoretically informative findings of this corpus and merits closer interpretation. Several non-exclusive explanations are plausible. First, the constructs may diverge: trait questionnaires such as the BIS, UPPS and I-7 aggregate impulsive behaviour across emotionally charged, real-world situations accumulated over time, whereas Stroop, stop-signal and antisaccade tasks index a narrow form of motor or interference control under emotionally neutral, highly structured laboratory conditions. Second, the discrepancy is consistent with a state-dependent account, in which impulse-control deficits in BPD emerge mainly under emotional load and are therefore largely absent from affectively neutral tasks; this is directly supported by the observation that inhibition errors increased under induced anxiety or anger only in the BPD group [25]. Third, the deficits that do appear under neutral conditions cluster in preparatory and anticipatory control rather than in the execution of inhibition, suggesting that what is altered in BPD is the readiness to deploy control in advance of a stimulus rather than the capacity to inhibit a response once required. On this reading, self-report instruments and laboratory tasks are not in conflict but are sensitive to different facets of impulsivity, and eye-tracking indices of preparatory control may capture an intermediate, mechanistically informative level that neither questionnaires nor classical accuracy-based inhibition tasks fully reflect.

### 4.3. Autonomic and Pupillary Indicators of Dysregulation

Physiological markers reinforce the picture of dysregulated arousal in BPD. Heart-rate variability indices (RMSSD, HF-HRV) were lower in BPD than in healthy controls [23], converging with meta-analytic evidence of reduced resting-state vagal tone in BPD [34]. Paediatric and adolescent BPD samples displayed larger baseline pupil sizes consistent with persistent sympathetic hyperarousal [28]. Pupillary reactivity to maternal criticism prospectively predicted increases in BPD symptoms over an 18-month follow-up [31]. Across peripheral, central and behavioural systems, then, BPD presents as a disorder of dysregulated arousal, in line with biosocial models that emphasise emotional vulnerability in interaction with invalidating environments [2] and with meta-analytic evidence implicating P300 alterations in BPD [10].

### 4.4. Modulation by Oxytocin and Emotional State

Pharmacological and contextual manipulations show that the social-attentional alterations in BPD can be modulated rather than being fixed traits. Intranasal oxytocin (24 to 26 IU) reduced amygdala and insula reactivity to social stimuli, attenuated the reflexive vigilance towards angry eyes, and brought BPD performance closer to that of healthy controls [17,18]. Conversely, induced anxiety or anger increased behavioural inhibition errors specifically in BPD [19]. These findings argue against a static trait conception of the BPD attentional profile and identify oxytocin as a candidate target with potential clinical relevance, complementing established psychotherapeutic approaches such as Dialectical Behaviour Therapy.

### 4.5. Integration with Prior Meta-Analytic and Neurobiological Evidence

The present synthesis is in line with previous meta-analytic work and adds further specificity. Kaiser et al. [6] reported an attentional pull towards emotional stimuli in BPD; the eye-tracking evidence reviewed here clarifies that this pull occurs in two stages and is feature-specific (eye region), and is increased by trauma history [24]. Domes et al. [12] documented alterations in facial emotion recognition in BPD; the studies reviewed here show that gaze deployment to ambiguous and neutral facial stimuli reflects a negative interpretation bias even when overt recognition deficits are absent [24,27]. The neuropsychological deficits identified by Ruocco [8] are matched, in the oculomotor domain, by the impairments in preparatory control discussed above, much of which seems attributable to comorbidity. The neurobiological model of Ruocco and Carcone [9], which emphasises reduced activation in the dorsal anterior cingulate, the inferior frontal gyrus and the inferior parietal sulcus, is consistent with the gaze–amygdala coupling findings reviewed here [17,31] and converges with multimodal neuroimaging meta-analyses identifying limbic hyperreactivity and prefrontal hypoactivation in BPD [36]. Only one previous systematic review of transdiagnostic eye-tracking biomarkers of inattention had included BPD, and only marginally [14]; the present BPD-specific synthesis therefore addresses a gap in the literature.

### 4.6. Limitations

The present synthesis has several limitations. Most of the seventeen included studies were cross-sectional case-control designs, with the majority involving adult female samples [17,18,19,20,21,22,23,24,25,26,27,32,33], which limits generalisability to males, gender-diverse populations and to the longitudinal course of BPD. This sex imbalance deserves emphasis rather than mere acknowledgement: thirteen of the seventeen studies recruited women only, so that the attentional, oculomotor and autonomic patterns described here are, strictly speaking, established for adult and adolescent women with BPD. Whether they generalise to men or to gender-diverse individuals with BPD is currently unknown, and statements about “patients with BPD” in this literature, including in the present review, should be read with that restriction in mind. Paediatric and adolescent evidence is restricted to three oculomotor studies [28,29,30] and to one developmental pupillometry cohort [31]. Methodological heterogeneity was also considerable: sampling rates ranged from 60 Hz to 500 Hz, area-of-interest definitions varied across studies, stimulus durations ran from 50 ms to 18 s, and calibration thresholds were not consistent. Beyond precluding quantitative meta-analysis, this heterogeneity constrains the strength of any mechanistic conclusion: because paradigms, stimulus sets, exposure durations, eye-tracking systems, metrics and operational definitions of the primary outcomes differ from study to study, apparently convergent findings may rest on non-equivalent operationalisations, and firm inferences about the cognitive or neural mechanisms underlying BPD cannot yet be drawn. A further interpretive limitation concerns ecological validity. Most paradigms in this corpus rely on static or briefly presented facial stimuli and on highly controlled laboratory tasks; although several studies deliberately incorporated more ecologically valid elements (for example, complex socio-emotional scenes, recordings of real maternal feedback, or competitive interactive games), the social demands of these tasks remain distant from naturalistic, reciprocal interaction, and the extent to which the patterns identified here generalise to real-world social functioning in BPD therefore remains uncertain. Psychiatric comorbidity (depression, anxiety, PTSD, ADHD) and psychotropic medication were prevalent and were controlled inconsistently across studies. The search strategy was restricted to three databases (Web of Science, Scopus, PubMed) and did not include PsycINFO; although the indexing overlap between these sources and the parallel artificial-intelligence-assisted cross-check make it unlikely that many eligible studies were missed, the omission of a psychology-specific database may nonetheless have introduced selection bias, and the grey literature was likewise not searched. Finally, the review was not preregistered and no a priori protocol was published, which reduces protection against post hoc changes to the eligibility criteria or synthesis plan and limits the extent to which selective decisions during the review can be excluded; we have therefore reported the eligibility rule, screening procedure and synthesis structure as transparently as possible, but acknowledge the absence of registration as a genuine limitation. At the level of the present manuscript, the absence of a quantitative synthesis with pooled effect sizes is itself a limitation, and a follow-up meta-analysis is justified once additional primary studies become available.

### 4.7. Clinical and Research Implications

From a clinical standpoint, the convergence of an early-vigilance and late-avoidance attentional profile, autonomic dysregulation and oxytocin-responsive amygdala reactivity supports the use of objective gaze and physiological measures as candidate markers for diagnosis, treatment monitoring and stratification in BPD. Eye-tracking metrics may complement self-report instruments and structured clinical interviews, and could help identify subgroups of patients in whom threat-related bias predominates over patterns of reduced engagement towards rewarding social cues. Treatments aimed at the underlying mechanisms, such as Dialectical Behaviour Therapy with its emphasis on distress tolerance and emotion regulation, oxytocin-based adjunctive approaches, or trauma-focused therapies such as EMDR [37], could incorporate eye-tracking outcomes as proximal indicators of change. Cochrane and focused systematic reviews have documented clinically meaningful reductions in BPD severity, self-harm and depression with these specialised psychotherapies relative to treatment as usual [38,39]. Beyond their cross-sectional descriptive value, the prospective findings of Scott et al. [31], in which pupillary reactivity to maternal criticism predicted increases in BPD symptoms over an 18-month interval, raise the possibility that these indices carry early-detection utility in at-risk adolescents before full clinical thresholds are reached. That the same indices are pharmacologically modifiable in adults [17,18] further suggests that they capture dynamic state–trait properties amenable to therapeutic targeting, rather than fixed neurocognitive deficits.

Three research priorities follow from this review. First, longitudinal designs are needed that track attentional and pupillary indices across symptom trajectories, particularly in adolescents at risk. Second, male and gender-diverse samples should be included, and standardised eye-tracking protocols with preregistered analytic plans should become the norm. Third, multimodal designs combining eye tracking with electrophysiology and functional neuroimaging, and transdiagnostic comparisons with Cluster-C personality disorders, PTSD and ADHD, are needed to delimit the specificity of the markers identified here.

### 4.8. Potential Clinical Applications and the Limits of Current Diagnostic Utility

Several concrete clinical applications can be envisaged for eye tracking and pupillometry in BPD, but it is important to separate what the present evidence supports in principle from what it can support in practice today. In principle, objective gaze and pupillary indices could serve three functions: as adjunctive markers that complement, rather than replace, structured clinical interviews; as stratification tools that distinguish patients in whom an early threat-related bias predominates from those characterised mainly by reduced engagement with rewarding cues; and as proximal outcome measures for monitoring change during interventions such as Dialectical Behaviour Therapy or oxytocin-based adjuncts. The prospective association between pupillary reactivity and later symptom change in at-risk adolescents [31] additionally raises the possibility of early-detection applications before full diagnostic thresholds are reached. At present, however, none of these applications are ready for clinical deployment. The diagnostic utility of the markers reviewed here is constrained by the absence of established sensitivity, specificity and predictive values for any individual index; by the lack of validated, standardised acquisition and scoring protocols across laboratories; by demonstrated sensitivity of the effects to comorbidity, medication and acute affective state; and by the near-total reliance on female samples. The group-level differences reported in this corpus, although reasonably consistent, are not the same as individual-level diagnostic accuracy, and no study in the corpus reported classification metrics suitable for diagnostic use. We therefore frame eye tracking and pupillometry in BPD as promising candidate markers for research and treatment monitoring, not as diagnostic tests; their translation into clinical practice will require dedicated diagnostic-accuracy studies with predefined thresholds, representative samples and external validation.

## 5. Conclusions

This systematic review of seventeen eye-tracking and physiological studies in borderline personality disorder identified a coherent set of findings: early reflexive vigilance to social cues, reduced engagement with positive stimuli during sustained viewing, autonomic dysregulation, and a discrepancy between high trait impulsivity on self-report and largely preserved laboratory inhibition. Comorbid PTSD, ADHD and psychotic-like symptoms moderated these patterns, and pharmacological challenge with intranasal oxytocin partly normalised amygdala–gaze coupling and attentional vigilance. The two-stage attentional profile is best summarised as a vigilance–avoidance pattern; it is consistent with the hypersensitivity hypothesis of BPD emotion dysregulation but does not establish it uniquely, since avoidance-based emotion regulation and reduced reward salience remain viable alternative accounts. Because thirteen of the seventeen studies recruited women only, these conclusions are established primarily for adult and adolescent women with BPD and should not yet be generalised to men or to gender-diverse populations. Overall, eye-tracking and pupillometric indices emerge as promising objective measures for clinical and research use rather than as validated diagnostic tools. Longitudinal, transdiagnostic and quantitative work, conducted with male and gender-diverse samples and standardised paradigms, and accompanied by formal diagnostic-accuracy evaluation, will be needed before these measures can be considered as actionable tools in the assessment, monitoring and treatment of BPD.

## Data Availability

No new primary data were generated in this study. All data analysed are derived from previously published primary studies cited in the reference list and are summarised in Table 1 and Table 2 of this manuscript. The full database search strategies used in Web of Science, Scopus, and PubMed are reported in Appendix A. Additional extraction details are available from the corresponding author upon reasonable request.

## Supplementary Materials

The following supporting information can be downloaded at https://www.mdpi.com/article/10.3390/brainsci16070712/s1, Table S1: the completed PRISMA 2020 checklist for this systematic review; Table S2: the full per-study methodological quality, risk-of-bias and ethical-transparency appraisal of the included studies (the extended version of Table 2). All data extracted from the included primary studies are presented in Table 1 and Table 2 of this manuscript. Database search strategies are detailed in Appendix A. The PRISMA 2020 flow diagram is shown in Figure 1.

## Abbreviations

The following abbreviations are used in this manuscript:

ACE	Adverse Childhood Experiences
ADHD	Attention-Deficit/Hyperactivity Disorder
AOI	Area of Interest
BDI-II	Beck Depression Inventory-II
BIS-11	Barratt Impulsivity Scale, 11-item
BPD	Borderline Personality Disorder
BSL-23	Borderline Symptom List, 23-item
CTQ	Childhood Trauma Questionnaire
DBT	Dialectical Behaviour Therapy
DERS	Difficulties in Emotion Regulation Scale
DSM-5	Diagnostic and Statistical Manual of Mental Disorders, 5th edition
ECG	Electrocardiogram
EDA	Electrodermal Activity
EOG	Electro-oculography
fMRI	Functional Magnetic Resonance Imaging
HC	Healthy Controls
HF-HRV	High-Frequency Heart-Rate Variability
HRV	Heart-Rate Variability
NR	Not Reported
OSF	Open Science Framework
PANAS	Positive and Negative Affect Schedule
PID-5	Personality Inventory for DSM-5
PRISMA	Preferred Reporting Items for Systematic Reviews and Meta-Analyses
PTSD	Post-Traumatic Stress Disorder
RMSSD	Root Mean Square of Successive Differences
RoB 2	Risk of Bias 2 tool for randomised trials
ROBINS-I	Risk Of Bias In Non-randomised Studies of Interventions
SCID	Structured Clinical Interview for DSM Disorders
SCR	Skin Conductance Response
STAI	State–Trait Anxiety Inventory

## Appendix A

Search strategies used in WoS, Scopus, and PubMed.

WoS: TS = ((“borderline personality disorder” OR “emotionally unstable personality disorder” OR EUPD OR “borderline personalit*” OR “borderline trait*” OR “borderline symptom*”))AND TS = ((“eye track*” OR gaze OR “visual scan*” OR fixation* OR saccad* OR antisaccad* OR pupil* OR oculomotor* OR “attentional bias” OR scanpath*))

Scopus: TITLE-ABS-KEY(“borderline personality disorder” OR “emotionally unstable personality disorder”

OR EUPD OR “borderline personalit*”

OR “borderline trait*”

OR “borderline symptom*”) AND TITLE-ABS-KEY (“eye track*” OR gaze OR “visual scan*”

OR fixation* OR saccad* OR antisaccad* OR pupil* OR oculomotor* OR “attentional bias” OR scanpath*)

PubMed: ((“Borderline Personality Disorder”[Mesh] OR “borderline personalit*”[tiab] OR “emotionally unstable personality disorder”[tiab] OR EUPD[tiab])

AND (“Eye Movements”[Mesh] OR “eye track*”[tiab] OR gaze[tiab] OR saccad*[tiab] OR fixation*[tiab] OR pupil*[tiab] OR oculomotor*[tiab] OR scanpath*[tiab] OR “attentional bias”[tiab]))

## References

[1] American Psychiatric Association (2013). Diagnostic and Statistical Manual of Mental Disorders.

[2] Bohus M., Stoffers-Winterling J., Sharp C., Krause-Utz A., Schmahl C., Lieb K. (2021). Borderline personality disorder. Lancet.

[3] Kjær J.N.R., Biskin R., Vestergaard C., Munk-Jørgensen P. (2020). All-cause mortality of hospital-treated borderline personality disorder: A nationwide cohort study. J. Pers. Disord..

[4] Ellison W.D., Rosenstein L.K., Morgan T.A., Zimmerman M. (2018). Community and clinical epidemiology of borderline personality disorder. Psychiatr. Clin. N. Am..

[5] Samuels J. (2011). Personality disorders: Epidemiology and public health issues. Int. Rev. Psychiatry.

[6] Kaiser D., Jacob G.A., Domes G., Arntz A. (2016). Attentional Bias for Emotional Stimuli in Borderline Personality Disorder: A Meta-Analysis. Psychopathology.

[7] Mesman J., van IJzendoorn M.H., Bakermans-Kranenburg M.J. (2009). The many faces of the still-face paradigm: A review and meta-analysis. Dev. Rev..

[8] Ruocco A.C. (2005). The neuropsychology of borderline personality disorder: A meta-analysis and review. Psychiatry Res..

[9] Ruocco A.C., Carcone D. (2016). A neurobiological model of borderline personality disorder: Systematic and integrative review. Harv. Rev. Psychiatry.

[10] Salas F., Nvo-Fernández M., Leiva-Bianchi M., Avello Sáez D., Sepúlveda Páez G., Via García M., Villacura-Herrera C. (2024). Components of event-related potentials and borderline personality disorder: A meta-analysis. Eur. J. Psychotraumatol..

[11] Holmqvist K., Nyström M., Andersson R. (2011). Eye Tracking: A Comprehensive Guide to Methods and Measures.

[12] Daros A.R., Zakzanis K.K., Ruocco A.C. (2013). Facial emotion recognition in borderline personality disorder. Psychol. Med..

[13] Arntz A., Bernstein D., Oorschot M., Schobre P. (2009). Theory of mind in borderline and Cluster-C personality disorder. J. Nerv. Ment. Dis..

[14] Toghi A., Mohammadzadeh A., Alemi Z., Khorrami Banaraki A. (2025). Transdiagnostic eye-tracking biomarkers of inattention across psychiatric disorders: A systematic review. BMC Psychiatry.

[15] Page M.J., McKenzie J.E., Bossuyt P.M., Boutron I., Hoffmann T.C., Mulrow C.D., Shamseer L., Tetzlaff J.M., Akl E.A., Brennan S.E. (2021). The PRISMA 2020 statement: An updated guideline for reporting systematic reviews. BMJ.

[16] Landis J.R., Koch G.G. (1977). The measurement of observer agreement for categorical data. Biometrics.

[17] Bertsch K., Gamer M., Schmidt B., Schmidinger I., Walther S., Kästel T., Schnell K., Büchel C., Domes G., Herpertz S.C. (2013). Oxytocin and Reduction of Social Threat Hypersensitivity in Women with Borderline Personality Disorder. Am. J. Psychiatry.

[18] Lischke A., Herpertz S.C., Berger C., Domes G., Gamer M. (2017). Divergent effects of oxytocin on (para-)limbic reactivity to emotional and neutral scenes in females with and without borderline personality disorder. Soc. Cogn. Affect. Neurosci..

[19] Jacob G.A., Gutz L., Bader K., Lieb K., Tüscher O., Stahl C. (2010). Impulsivity in Borderline Personality Disorder: Impairment in Self-Report Measures, but Not Behavioral Inhibition. Psychopathology.

[20] Bertsch K., Krauch M., Stopfer K., Haeussler K., Herpertz S.C., Gamer M. (2017). Interpersonal Threat Sensitivity in Borderline Personality Disorder: An Eye-Tracking Study. J. Pers. Disord..

[21] Seitz K.I., Leitenstorfer J., Krauch M., Hillmann K., Boll S., Ueltzhoeffer K., Neukel C., Kleindienst N., Herpertz S.C., Bertsch K. (2021). An eye-tracking study of interpersonal threat sensitivity and adverse childhood experiences in borderline personality disorder. Borderline Personal. Disord. Emot. Dysregul..

[22] Niedtfeld I., Renkewitz F., Mädebach A., Hillmann K., Kleindienst N., Schmahl C., Schulze L. (2020). Enhanced Memory for Negative Social Information in Borderline Personality Disorder. J. Abnorm. Psychol..

[23] Bortolla R., Cavicchioli M., Galli M., Verschure P.F.M.J., Maffei C. (2019). A comprehensive evaluation of emotional responsiveness in borderline personality disorder: A support for hypersensitivity hypothesis. Borderline Personal. Disord. Emot. Dysregul..

[24] Bortolla R., Galli M., Ramella P., Sirtori F., Visintini R., Maffei C. (2020). Negative bias and reduced visual information processing of socio-emotional context in borderline personality disorder: A support for the hypersensitivity hypothesis. J. Behav. Ther. Exp. Psychiatry.

[25] Bortolla R., Spada G.E., Lazzarino E., Maffei C. (2023). Eye-tracking patterns in borderline personality disorder: Findings from a relational dot-probe task. Mediterr. J. Clin. Psychol..

[26] Wenk T., Günther A.C., Webelhorst C., Kersting A., Bodenschatz C.M., Suslow T. (2024). Reduced positive attentional bias in patients with borderline personality disorder compared with non-patients: Results from a free-viewing eye-tracking study. Borderline Personal. Disord. Emot. Dysregul..

[27] Wenk T., Bartusch M., Webelhorst C., Kersting A., Bodenschatz C.M., Suslow T. (2025). Gaze and Evaluative Behavior of Patients with Borderline Personality Disorder in an Affective Priming Task. Behav. Sci..

[28] Calancie O.G., Parr A.C., Brien D.C., Huang J., Pitigoi I.C., Coe B.C., Booij L., Khalid-Khan S., Munoz D.P. (2023). Motor synchronization and impulsivity in pediatric borderline personality disorder with and without attention-deficit hyperactivity disorder: An eye-tracking study of saccade, blink and pupil behavior. Front. Neurosci..

[29] Calancie O.G., Parr A.C., Brien D.C., Coe B.C., Booij L., Khalid-Khan S., Munoz D.P. (2024). Impairment of Visual Fixation and Preparatory Saccade Control in Borderline Personality Disorder with and Without Comorbid Attention-Deficit/Hyperactivity Disorder. Biol. Psychiatry Cogn. Neurosci. Neuroimaging.

[30] Parr A.C., Calancie O.G., Coe B.C., Khalid-Khan S., Munoz D.P. (2022). Impulsivity and Emotional Dysregulation Predict Choice Behavior During a Mixed-Strategy Game in Adolescents with Borderline Personality Disorder. Front. Neurosci..

[31] Scott L.N., Zalewski M., Beeney J.E., Jones N.P., Stepp S.D. (2017). Pupillary and affective responses to maternal feedback and the development of borderline personality disorder symptoms. Dev. Psychopathol..

[32] Grootens K.P., van Luijtelaar G., Buitelaar J.K., van der Laan A., Hummelen J.W., Verkes R.J. (2008). Inhibition errors in borderline personality disorder with psychotic-like symptoms. Prog. Neuro-Psychopharmacol. Biol. Psychiatry.

[33] Kaiser D., Jacob G.A., van Zutphen L., Siep N., Sprenger A., Tuschen-Caffier B., Senft A., Arntz A., Domes G. (2019). Biased Attention to Facial Expressions of Ambiguous Emotions in Borderline Personality Disorder: An Eye-Tracking Study. J. Pers. Disord..

[34] Koenig J., Kemp A.H., Feeling N.R., Thayer J.F., Kaess M. (2016). Resting state vagal tone in borderline personality disorder: A meta-analysis. Prog. Neuropsychopharmacol. Biol. Psychiatry.

[35] Bar-Haim Y., Lamy D., Pergamin L., Bakermans-Kranenburg M.J., Van IJzendoorn M.H. (2007). Threat-related attentional bias in anxious and nonanxious individuals: A meta-analytic study. Psychol. Bull..

[36] Schulze L., Schmahl C., Niedtfeld I. (2016). Neural correlates of disturbed emotion processing in borderline personality disorder: A multimodal meta-analysis. Biol. Psychiatry.

[37] Nvo-Fernández M., Salas F., Miño-Reyes V., Ahumada-Méndez F., Medina P., Avello D., Floriano Landim S., Via M., Napolitano N., Leiva-Bianchi M. (2025). Effectiveness of Eye Movement Desensitization and Reprocessing (EMDR) in Treating Borderline Personality Disorder: A Randomized Controlled Trial. Alpha Psychiatry.

[38] Storebø O.J., Stoffers-Winterling J.M., Völlm B.A., Kongerslev M.T., Mattivi J.T., Jørgensen M.S., Faltinsen E., Todorovac A., Sales C.P., Callesen H.E. (2020). Psychological therapies for people with borderline personality disorder. Cochrane Database Syst. Rev..

[39] Stoffers-Winterling J.M., Storebø O.J., Kongerslev M.T., Faltinsen E., Todorovac A., Jørgensen M.S., Sales C.P., Callesen H.E., Pereira Ribeiro J., Völlm B.A. (2022). Psychotherapies for borderline personality disorder: A focused systematic review and meta-analysis. Br. J. Psychiatry.

