# Supplementary material for: Eye-Tracking and Borderline Personality Disorder: A Systematic Review
Source: Brain Sci. 2026 Jul 1;16(7):712. doi: 10.3390/brainsci16070712 (PMC13406454; doi:10.3390/brainsci16070712)
Supplement: Supplementary file 1 [file brainsci-16-00712-s001.zip › Supplementary_Table_S1_PRISMA_Checklist-1.pdf]

Table S1. PRISMA 2020 Checklist

Eye-Tracking and Borderline Personality Disorder: A Systematic Review

Leiva-Bianchi, M., & Nvo-Fernández, M. — Brain Sciences (2026)

Reference: Page MJ, McKenzie JE, Bossuyt PM, Boutron I, Hoffmann TC, Mulrow CD, et al. The PRISMA 2020 statement: an updated guideline for reporting systematic reviews. *BMJ* 2021;372:n71. https://doi.org/10.1136/bmj.n71.

Section and Topic	Item #	Checklist item	Location where item is reported
**TITLE**			
Title	1	Identify the report as a systematic review.	Title — "Eye-Tracking and Borderline Personality Disorder: A Systematic Review".
**ABSTRACT**			
Abstract	2	See the PRISMA 2020 for Abstracts checklist.	Abstract, structured under Background/Objectives, Methods, Results and Conclusions.
**INTRODUCTION**			
Rationale	3	Describe the rationale for the review in the context of existing knowledge.	Section 1 (Introduction), paragraphs 1–3.
Objectives	4	Provide an explicit statement of the objective(s) or question(s) the review addresses.	Section 1 (Introduction), final paragraph.
**METHODS**			
Eligibility criteria	5	Specify the inclusion and exclusion criteria for the review and how studies were grouped for the syntheses.	Section 2 (Materials and Methods); grouping also reflected in the Section 3 subheadings.
Information sources	6	Specify all databases, registers, websites, organisations, reference lists and other sources searched or consulted to identify studies. Specify the date when each source was last searched or consulted.	Section 2: Web of Science, Scopus, PubMed, searched up to 13 March 2026.
Search strategy	7	Present the full search strategies for all databases, registers and websites, including any filters and limits used.	Appendix A (full search strategies for WoS, Scopus, PubMed).
Selection process	8	Specify the methods used to decide whether a study met the inclusion criteria of the review, including how many reviewers screened each record and each report retrieved, whether they worked independently, and if applicable, details of automation tools used in the process.	Section 2: two independent reviewers; Cohen's $\kappa = 1.00$ at title-and-abstract stage; AI-assisted check described as non-systematic and not part of the search.

Section and Topic	Item #	Checklist item	Location where item is reported
Data collection process	9	Specify the methods used to collect data from reports, including how many reviewers collected data from each report, whether they worked independently, any processes for obtaining or confirming data from study investigators, and if applicable, details of automation tools used in the process.	Section 2: independent extraction by one reviewer and verification by the other; discrepancies resolved by consensus; no automation tools used for extraction.
Data items	10a	List and define all outcomes for which data were sought. Specify whether all results that were compatible with each outcome domain in each study were sought, and if not, the methods used to decide which results to collect.	Section 3.3 (Primary and Secondary Outcomes); Table 1 (Outcomes; Instruments or measures).
Data items	10b	List and define all other variables for which data were sought. Describe any assumptions made about any missing or unclear information.	Sections 3.1 and 3.2; Tables 1 and 2; missing items recorded explicitly as “NR”.
Study risk of bias assessment	11	Specify the methods used to assess risk of bias in the included studies, including details of the tool(s) used, how many reviewers assessed each study and whether they worked independently, and if applicable, details of automation tools used in the process.	Section 2: RoB 2 for randomised controlled trials and Newcastle–Ottawa Scale logic for observational studies (ROBINS-I held in reserve but not applied); per-study framework in Table 2; harmonised four-level mapping rule reported in Section 2.
Effect measures	12	Specify for each outcome the effect measure(s) used in the synthesis or presentation of results.	Section 2: no quantitative pooling; effect estimates reported verbatim from individual studies in Tables 1 and 2.
Synthesis methods	13a	Describe the processes used to decide which studies were eligible for each synthesis.	Section 2: grouping by outcome family and by pharmacological / interpersonal-stress manipulation.
Synthesis methods	13b	Describe any methods required to prepare the data for presentation or synthesis.	Section 2: no recomputation of effect sizes; estimates reported verbatim.
Synthesis methods	13c	Describe any methods used to tabulate or visually display results of individual studies and syntheses.	Tables 1 and 2; narrative synthesis in Sections 3.6–3.10.
Synthesis methods	13d	Describe any methods used to synthesise results and provide a rationale for the choice(s). If meta-analysis was performed, describe the model(s) and software.	Section 2: narrative synthesis only, justified by methodological heterogeneity.

Section and Topic	Item #	Checklist item	Location where item is reported
Synthesis methods	13e	Describe any methods used to explore possible causes of heterogeneity among study results.	Section 2: qualitative inspection of paradigm, sample and outcome characteristics; discussed in Sections 3.6–3.10 and 4.
Synthesis methods	13f	Describe any sensitivity analyses conducted to assess robustness of the synthesised results.	Not applicable; no quantitative pooling was conducted.
Reporting bias assessment	14	Describe any methods used to assess risk of bias due to missing results in a synthesis (arising from reporting biases).	Section 2 (final methods paragraph): formal funnel-plot assessment not feasible; small samples, infrequent preregistration and exploratory analyses noted as elevating risk.
Certainty assessment	15	Describe any methods used to assess certainty (or confidence) in the body of evidence for an outcome.	Section 2 (GRADE paragraph): GRADE not performed; per-study quality on a four-level scale in Table 2; qualitative strength-of-evidence statement provided.
**RESULTS**			
Study selection	16a	Describe the results of the search and selection process, from the number of records identified to the number of studies included, ideally using a flow diagram.	Figure 1 (PRISMA flow diagram); Section 2 (selection process).
Study selection	16b	Cite studies that might appear to meet the inclusion criteria, but which were excluded, and explain why they were excluded.	Not applicable: all seventeen reports retrieved at the full-text stage met the inclusion criteria, and no report was excluded at the full-text eligibility stage (Figure 1; Section 2).
Study characteristics	17	Cite each included study and present its characteristics.	Table 1 (with each study cited by reference number, e.g. [1]–[17]); Sections 3.1 and 3.2.
Risk of bias in studies	18	Present assessments of risk of bias for each included study.	Table 2 (Quality appraisal column, framework declared per study); Section 3.4.
Results of individual studies	19	For all outcomes, present, for each study, summary statistics for each group (where appropriate) and an effect estimate and its precision, ideally using structured tables or plots.	Table 2 (Main findings column) — test statistics, p values and effect sizes reported verbatim where available.
Results of syntheses	20a	For each synthesis, briefly summarise the characteristics and risk of bias among contributing studies.	Sections 3.6–3.10; cross-referenced with Tables 1 and 2.
Results of syntheses	20b	Present results of all statistical syntheses conducted. If meta-analysis was done, present summary estimates and measures of heterogeneity.	Not applicable; no statistical syntheses were conducted.
Results of syntheses	20c	Present results of all investigations of possible causes of heterogeneity among study results.	Sections 3.6–3.10 and Section 4 (Discussion).

Section and Topic	Item #	Checklist item	Location where item is reported
Results of syntheses	20d	Present results of all sensitivity analyses conducted to assess robustness.	Not applicable; no quantitative synthesis.
Reporting biases	21	Present assessments of risk of bias due to missing results (arising from reporting biases) for each synthesis assessed.	Section 2 (final methods paragraph); discussed in Section 4.6 (Limitations).
Certainty of evidence	22	Present assessments of certainty (or confidence) in the body of evidence for each outcome assessed.	Section 2 (GRADE paragraph); per-study quality in Table 2; qualitative strength-of-evidence statement in Section 2.
**DISCUSSION**			
Discussion	23a	Provide a general interpretation of the results in the context of other evidence.	Sections 4.1–4.5 (Discussion, including integration with prior evidence).
Discussion	23b	Discuss any limitations of the evidence included in the review.	Section 4.6 (Limitations).
Discussion	23c	Discuss any limitations of the review processes used.	Section 4.6 (Limitations) — three databases, PsycINFO not searched, no quantitative synthesis, no registration.
Discussion	23d	Discuss implications of the results for practice, policy, and future research.	Sections 4.7 and 4.8 (Clinical and Research Implications; Diagnostic Utility); Section 5 (Conclusions).
**OTHER INFORMATION**			
Registration and protocol	24a	Provide registration information for the review, including the register name and registration number, or state that the review was not registered.	Section 2: the review was not registered in PROSPERO or any other registry; consequently, no registration number is available.
Registration and protocol	24b	Indicate where the review protocol can be accessed, or state that a protocol was not prepared.	Section 2: no a priori protocol was prepared or published.
Registration and protocol	24c	Describe and explain any amendments to information provided at registration or in the protocol.	Not applicable (no registration or protocol).
Support	25	Describe sources of financial or non-financial support for the review, and the role of the funders or sponsors in the review.	Funding statement: “This research received no external funding.”
Competing interests	26	Declare any competing interests of review authors.	Conflicts of Interest statement: the authors declare no conflicts of interest.
Availability of data, code	27	Report which of the following are publicly available and where they can	Data Availability Statement: all extracted data are presented in Tables 1 and 2; search

Section and Topic	Item #	Checklist item	Location where item is reported
and other materials		be found: data collection forms; data extracted from included studies; data used for all analyses; analytic code; any other materials.	strategies in Appendix A; further details available from the corresponding author on reasonable request.

Note. Section numbers refer to the submitted manuscript. Items 13f, 16b, 20b and 20d are marked "Not applicable": the methodological heterogeneity of the included studies precluded quantitative pooling (13f, 20b, 20d), and no report was excluded at the full-text eligibility stage (16b). The review was not registered and no a priori protocol was prepared (items 24a–24c); this is stated transparently in Section 2 and acknowledged as a limitation in Section 4.6.
