# Supplementary material for: Eye-Tracking and Borderline Personality Disorder: A Systematic Review
Source: Brain Sci. 2026 Jul 1;16(7):712. doi: 10.3390/brainsci16070712 (PMC13406454; doi:10.3390/brainsci16070712)
Supplement: Supplementary file 1 [file brainsci-16-00712-s001.zip › Supplementary_Table_S2_Full_Appraisal-1.pdf]

Table S2. Full per-study methodological quality, risk-of-bias and ethical-transparency appraisal

Eye-Tracking and Borderline Personality Disorder: A Systematic Review

Leiva-Bianchi, M., & Nvo-Fernández, M. — Brain Sciences (2026). This table provides the complete appraisal summarised in Table 2 of the main text.

Study	Quality appraisal of the study	Risk of bias / quality notes	Funding / conflicts of interest	Ethical considerations	Main findings and conclusion
Bertsch et al. 2013 [2]	Framework used: RoB 2 Randomization process: Reported randomized assignment. Method: NR. Allocation concealment: Double-blind placebo design suggests adequate concealment. Details: NR. Blinding: Participants and investigators blinded. Missing outcome data: Behavioral data excluded in 13 participants due to artifacts. Outcome measurement: Objective eye-tracking and fMRI measures. Selective reporting: No clear evidence of selective reporting. Overall quality judgment: Some concerns.	Small sample. Female only. Behavioral data exclusions. No clinical control group	German Federal Ministry for Education and Research grant. No financial conflicts reported	Ethics approval: Yes (Heidelberg Faculty of Medicine) Informed consent: Written Vulnerable populations: Psychiatric patients Risk–benefit balance: Minimal pharmacological risk Privacy/data protection: NR Compensation: Financial Trial registration: NR Protocol deviations: Behavioral data exclusions Data availability: NR Preregistration: NR Selective reporting risk: Moderate Generative AI use: NR Overall ethical transparency judgment: Adequate	BPD placebo group showed faster fixation changes and increased amygdala activation to angry faces. Oxytocin reduced fixation changes toward angry eyes and reduced right posterior amygdala activation. Borderline patients responded faster to angry faces vs controls (F=3.94, p=0.03). Significant interaction for fixation changes (F=4.02, p=0.02). Conclusion: Oxytocin reduces social threat hypersensitivity and amygdala hyperreactivity in female BPD patients
Bertsch et al. 2017 [3]	Study type: Laboratory experimental case–control study Framework used: Newcastle-Ottawa logic (observational experimental) Selection bias: Clinical sample with comorbidities. Female only. Confounding: Some covariate control (depression. anxiety). No clinical control group. Exposure classification: Clear diagnostic interviews. Outcome measurement quality: Objective eye-tracking measures. Missing data: Substantial reduction for saccade latency analyses. Selective reporting: Unclear. Multiple exploratory analyses. Overall quality judgment: Moderate.	Small sample. Female only. Comorbidities. Subsample for saccade latency analyses	German Research Foundation. European Research Council. No conflicts declared	Ethics approval: Yes (local university ethics committee) Informed consent: Written Vulnerable populations: Psychiatric patients Risk–benefit balance: Minimal Privacy/data protection: NR Compensation: NR Trial registration: NR Protocol deviations: NR Data availability: NR Preregistration: NR Selective reporting risk: Moderate Generative AI use: NR Overall ethical transparency judgment: Limited	BPD patients slower overall responses (F=5.79, p=.012). Misclassified faces as angry more often in brief condition (interaction F=3.98, p=.012). Faster initial saccades toward eyes of neutral faces and slower disengagement from fearful eyes in brief condition. No group differences in fixation duration overall Conclusion: BPD shows specific early bias toward interpersonal threat cues and deficits in detailed processing linked to aggressiveness
Bortolla et al. 2019 [7]	Study type: Observational laboratory experimental case–control Framework used: Newcastle-Ottawa logic Selection bias: Small clinical outpatient sample. Mixed medication status. Confounding: Limited control for comorbidities and medication. Exposure classification: Structured diagnostic interview. Outcome measurement: Objective multimodal measures. Missing data: Loss of EDA data in ~36% of BPD group. Selective reporting: Extensive exploratory analyses. Overall quality judgment: Moderate.	Small sample. medication use allowed. missing EDA data. no clinical control group	NR	Ethics approval: NR Informed consent: Written Vulnerable populations: Psychiatric patients Risk–benefit balance: Minimal Privacy/data protection: NR Compensation: NR Clinical trial registration: NR Protocol deviations: Technical data loss (EDA) Data availability: NR Preregistration: NR Selective reporting risk: Moderate Generative AI use: NR Overall ethical transparency judgment: Limited	BPD showed faster SCR latency (F=4.39, p<.05, η^2=.17). Reduced visual exploration of socio-emotional cues (prop gaze F=5.73, p<.05; fixation duration F=7.64, p<.05). No hyperreactivity effects. Significant group×block interaction for valence ratings (F=13.54, p<.01) and RMSSD (F=10.05, p<.01) indicating impaired emotional modulation Conclusion: Results support hypersensitivity and slow return to baseline but not hyperreactivity hypotheses
Bortolla et al. 2020 [8]	Study type: Laboratory experimental case–control Framework used: Newcastle-Ottawa logic Selection bias: Small outpatient sample. Female only. Medication allowed. Confounding: Limited control of depression and anxiety state effects. Exposure classification: Structured DSM-5 interview. Outcome measurement: Objective eye-tracking and validated	Female-only sample. high comorbidity and medication use. no clinical control group	No specific funding. No competing interests declared	Ethics approval: NR Informed consent: Written Vulnerable populations: Psychiatric patients Risk–benefit balance: Minimal Privacy/data protection: NR Compensation: NR Clinical trial registration: NR Protocol deviations: NR Data availability: NR Preregistration: NR Selective reporting risk: Moderate Generative AI use: NR Overall ethical transparency judgment: Limited	BPD rated socio-emotional stimuli more negatively (F=5.04. p=.03). Reduced number of fixations (F=11.74. p=.002) and reduced time in AoIs (F=9.07. p=.005). Interaction effects showed reduced exploration especially for negative and neutral stimuli and during prolonged exposure (18 s block). Conclusion: Emotional dysregulation in BPD reflects negative appraisal bias and reduced social information processing rather than hyperreactivity

Study	Quality appraisal of the study	Risk of bias / quality notes	Funding / conflicts of interest	Ethical considerations	Main findings and conclusion
	affective scales. Missing data: Not substantial for main outcomes. Selective reporting: Multiple exploratory analyses and interactions. Overall quality judgment: Moderate.				
Bortolla et al. 2023 [9]	Study type: Laboratory experimental case–control Framework used: Newcastle-Ottawa logic Selection bias: Small outpatient sample. mixed gender but mostly female. Confounding: Psychiatric comorbidities and medication allowed. Exposure classification: Structured DSM-5 diagnostic interview. Outcome measurement: Objective eye-tracking and standardized dot-probe metrics. Missing data: Not substantial. Selective reporting: Multiple exploratory correlations and interaction analyses. Overall quality judgment: Moderate.	Medication use common. comorbidity present. no clinical control group	No conflicts declared. Funding NR	Ethics approval: Compliance with APA ethical standards Informed consent: Written Vulnerable populations: Psychiatric patients Risk–benefit balance: Minimal Privacy/data protection: NR Compensation: NR Clinical trial registration: NR Protocol deviations: NR Data availability: On request Preregistration: NR Selective reporting risk: Moderate Generative AI use: NR Overall ethical transparency judgment: Limited	Higher bias score for erotic stimuli and lower bias score for negative stimuli in BPD (group×condition F=3.25, p=.05). Reduced latency to erotic and negative stimuli indicating hypervigilance. Reduced visual exploration of negative stimuli (less time and duration). Higher baseline negative affect in BPD (F=8.68, p=.005). Conclusion: BPD shows hypervigilance to relational stimuli with content-specific later attentional patterns and modulation by baseline negative affect
Calancie et al. 2023 [12]	Study type: Laboratory experimental case–control Framework used: Newcastle-Ottawa logic Selection bias: Clinical outpatient sample. female-only. Confounding: ADHD comorbidity and psychotropic medication common. Exposure classification: Structured diagnostic interview. Outcome measurement: High-precision eye-tracking. objective metrics. Missing data: Minimal reporting. Selective reporting: Multiple exploratory analyses. Overall quality judgment: Moderate.	Medication use allowed. only females. comorbidities present	NR	Ethics approval: Institutional review procedures reported Informed consent: Parent consent and youth assent Vulnerable populations: Minors with psychiatric disorders Risk–benefit balance: Minimal behavioral task risk Privacy/data protection: NR Compensation: NR Trial registration: NR Protocol deviations: NR Data availability: NR Preregistration: NR Selective reporting risk: Moderate Generative AI use: NR Overall ethical transparency judgment: Limited	No group differences in temporal prediction during predictable condition. ADHD/BPD showed more anticipatory saccades in random condition (χ^2=11.13, p=.004). ADHD/BPD showed higher blink rate (p<;.001). Both clinical groups showed larger pupil size vs controls (p<;.001). Conclusion: Temporal motor prediction intact in BPD. response inhibition deficits mainly linked to ADHD comorbidity. increased sympathetic arousal in BPD.
Calancie et al. 2024 [13]	Study type: Laboratory experimental case–control Framework used: Newcastle-Ottawa logic Selection bias: Clinical outpatient adolescent sample. female only. Confounding: High psychiatric comorbidity and medication exposure. Exposure classification: Structured diagnostic assessment. Outcome measurement: High-precision objective eye-tracking metrics. Missing data: Minimal. preprocessing success >;96%. Selective reporting: Multiple exploratory outcomes. Overall quality judgment: Moderate.	Female-only adolescent sample. comorbidities allowed. medication status not fully controlled	Canadian Institutes of Health Research; SEAMO AFP Innovation Fund. No conflicts declared	Ethics approval: Yes (Queen’s University protocol) Informed consent: Written consent or parental consent with youth assent Vulnerable populations: Minors with psychiatric disorders Risk–benefit balance: Minimal behavioral task risk Privacy/data protection: NR Compensation: Financial reimbursement Clinical trial registration: NR Protocol deviations: NR Data availability: NR Preregistration: NR Selective reporting risk: Moderate Generative AI use: NR Overall ethical transparency judgment: Adequate	Reduced fixation acquisition in BPD and ADHD/BPD vs controls (χ^2=13.81, p=.001). Increased fixation breaks (χ^2=22.65, p ≈ 1.2×10^{-5}). ADHD/BPD showed more anticipatory saccades (χ^2=11.65, p=.003) and higher antisaccade error rate (~32.7% vs 19.6% controls). Greater variability of saccade RT (χ^2=22.78, p ≈ 1.1×10^{-5}). No group differences in median RT or voluntary override time. Conclusion: BPD involves impaired preparatory oculomotor control; additional response-inhibition deficits linked to ADHD comorbidity.
Grootens et al. 2008 [15]	Study type: Laboratory experimental case–control Framework used: Newcastle-Ottawa logic Selection bias: Clinical heterogeneous sample. psychosis subgroup defined retrospectively. Confounding: Medication exposure and sex imbalance. Exposure classification: Structured diagnostic interviews. Outcome measurement: Objective EOG-based eye-movement	Medication use allowed; unequal sex distribution; heterogeneous clinical sample	NR	Ethics approval: Yes (local ethics committee) Informed consent: Written Vulnerable populations: Psychiatric patients Risk–benefit balance: Minimal Privacy/data protection: NR Compensation: €10/hour Clinical trial registration: NR Protocol deviations: NR Data availability: NR Preregistration: NR Selective reporting risk: Moderate Generative AI use: NR Overall	Significant group effect for inhibition errors (F=10.3, p<;.001). Schizophrenia >; BPD >; controls. BPD with psychotic-like symptoms showed more inhibition errors than BPD without (F=10.2, p<;.01). BPD had more anticipatory errors than controls (F=4.60, p<;.05). Conclusion: Inhibition deficits characterize a subgroup of BPD patients with psychotic-like symptoms and resemble schizophrenia patterns.

Study	Quality appraisal of the study	Risk of bias / quality notes	Funding / conflicts of interest	Ethical considerations	Main findings and conclusion
	measures. Missing data: Some unusable trials. not excessive. Selective reporting: Focused primary hypothesis. Overall quality judgment: Moderate.			ethical transparency judgment: Adequate	
Jacob et al., 2010 [1]	Study type: Cross-sectional observational laboratory study Framework used: Newcastle-Ottawa logic (adapted) Key strengths: Careful pairwise matching on age and education. Structured diagnostic interviews (SCID-I and II). Multiple validated impulsivity measures Behavioral paradigms with established reliability Key limitations: Very small sample size. Female-only sample limits generalizability. Lifetime psychiatric comorbidity present. Cross-sectional design prevents causal inference. Risk of bias or quality concerns Selection bias possible: Residual confounding. Limited statistical power Overall quality judgment: Moderate (Fair)	Small sample. Female-only sample. Possible residual confounding from lifetime comorbidity	NR	Ethics approval: Yes. Local ethics board University of Freiburg Informed consent: Written consent obtained Vulnerable populations: Psychiatric patients included. Safeguards not detailed Risk–benefit balance: Minimal risk cognitive tasks Privacy and data protection: NR Compensation: NR Trial registration: Not applicable Protocol deviations: NR Data availability: NR Preregistration: NR Selective reporting risk: Possible due to multiple outcomes Generative AI use: NR Overall ethical transparency judgment: Limited	Higher impulsivity in BPD on most self-report scales (e.g., BIS behavioral mean 2.5 vs 1.9, p<0.001). No significant group differences in stop signal or antisaccade tasks. Stroop difference small and nonsignificant after correction Conclusion: BPD shows elevated self-reported impulsivity but not clear deficits in laboratory behavioral inhibition. Emotional state may modulate performance
Kaiser et al., 2019 [16]	Study type: Cross-sectional observational laboratory study Framework used: Newcastle-Ottawa logic Key strengths: Use of objective eye-tracking measure. Clinical comparison group (Cluster-C PD). Examination of PTSD subgroup Key limitations: Full methodological details unavailable. Sample size unknown Statistical approach unclear. Risk of bias or quality concerns: Selection bias unclear. Measurement validity cannot be evaluated. Reporting bias possible Overall quality judgment: Unclear / likely Moderate-to-Low confidence (due to missing data)	High uncertainty due to missing methodological details	NR	NR	BPD patients fixated eyes longer than non-patients for angry/happy, sad/happy, fearful/sad blends. Effect mainly driven by BPD with PTSD Conclusion: Attention bias toward eyes in ambiguous emotions in BPD may be trauma-related rather than due to emotion recognition deficits
Lischke et al., 2017 [5]	Study type: Laboratory experimental intervention Framework used: RoB 2 Key strengths: Double-blind design. Large sample for fMRI BPD research Hormonal cycle control. Multimodal outcomes (behavior + eye-tracking + neuroimaging). Key limitations: Between-subjects drug allocation (no crossover). Female-only sample. Behavioral ceiling effects. Short-term administration only. Risk of bias or quality concerns: Possible residual confounding. Limited ecological validity. Overall quality judgment: Moderate-to-Good	Female-only sample. Single-dose design. Behavioral ceiling effects	Funded by German Federal Ministry of Education and Research. European Research Council. German Research Foundation. No conflicts declared	Ethics approval: Local ethics committee Informed consent: Written Vulnerable populations: Psychiatric patients. Safeguards not detailed Risk–benefit balance: Low risk neuroimaging + pharmacological challenge Privacy/data protection: NR Compensation: NR Trial registration: NR Protocol deviations: Technical exclusions reported Data availability: Supplementary material referenced Preregistration: NR Selective reporting risk: Possible Generative AI use: NR Overall ethical transparency judgment: Adequate	After placebo: BPD showed increased amygdala (Z=3.99 PFWE=0.006) and insula activity vs HC. Oxytocin decreased amygdala/insula reactivity in BPD but increased it in HC (interaction Z≈3.67–3.74 PFWE≈0.013–0.016). No group differences in task accuracy or fix ation number Conclusion: Oxytocin normalizes hyperreactive paralimbic responses and abnormal neural–gaze coupling in BPD during scene processing
Niedtfeld et al., 2020 [4]	Study type: Cross-sectional experimental cognitive study Framework used: Newcastle-Ottawa logic Key strengths: Adequate clinical sample. Rigorous diagnostic interviews. Novel validated paradigm. Open science data sharing. Key limitations:	NR	German Research Foundation grants	Ethics approval: Ethics approval Heidelberg Medical Faculty (protocol 2013-654N-MA) Informed consent: Written Vulnerable populations: Psychiatric patients included Risk–benefit balance: Minimal cognitive task risk Privacy/data	Generalized linear mixed-effects models. Linear mixed models. Correlation analyses Conclusion: Enhanced retrieval of negative person information may reinforce dysfunctional schemas and interpersonal distrust in BPD

Study	Quality appraisal of the study	Risk of bias / quality notes	Funding / conflicts of interest	Ethical considerations	Main findings and conclusion
	Female-only sample. No psychiatric control group. Eye-tracking reliability limitations. Possible salience vs valence confounding. Risk of bias or quality concerns. Residual confounding (comorbidities). Selection bias possible Overall quality judgment: Moderate (Fair-Good)			protection: NR Compensation: NR Trial registration: NR Protocol deviations: Exclusions reported Data availability: Materials and dataset publicly shared (OSF) Preregistration: NR Selective reporting risk: Moderate Generative AI use: NR Overall ethical transparency judgment: Adequate	
Parr et al., 2022 [17]	Study type: Laboratory experimental case-control study. Framework used: Newcastle-Ottawa logic. Key strengths: Structured DSM-5 diagnostic interview (SCID-5-PD). Sex- and age-matched controls. High-precision monocular eye-tracking at 500 Hz. Multiple oculomotor and behavioral outcomes. Key limitations: Female-only adolescent sample. Medication regimen not interrupted (mixed psychotropic exposure). Cross-sectional design. Exploratory study with multiple comparisons. Risk of bias or quality concerns: Selection bias possible (single-site recruitment from DBT outpatient clinic). Residual confounding from comorbid ADHD. Overall quality judgment: Moderate (Fair-Good).	Female-only adolescent sample. Medication regimen not interrupted. Single-site recruitment. Comorbid ADHD common in BPD subgroup.	No specific funding statement reported. Authors declared no conflicts of interest.	Ethics approval: Yes (Queen's University Human Research Ethics Board). Informed consent: Written consent for participants ≥17 years; oral assent and parental written consent for participants 11–16 years. Vulnerable populations: Minors with psychiatric disorders. Risk-benefit balance: Minimal behavioral task risk. Privacy/data protection: NR. Compensation: $30 gift card and rate of $20/hour. Trial registration: NR. Protocol deviations: One BPD participant excluded for poor task understanding. Data availability: NR. Preregistration: NR. Selective reporting risk: Moderate (multiple exploratory analyses). Generative AI use: NR. Overall ethical transparency judgment: Adequate.	BPD adolescents showed greater percentage of anticipatory saccadic decisions vs controls (M = 6.71% vs 3.32%, β = -0.92, t = -3.73, p < 0.001 Bonferroni-corrected). Increased coefficient of variation in saccadic reaction time (M = 32.88% vs 29.72%, p = 0.05 uncorrected). Reduced lose-shift bias in color domain (β = 0.62, t = 2.31, p = 0.03). Higher Barratt Impulsivity Scale scores (β = -1.28, t = -5.87, p < 0.001). Within-group: BPD participants with higher motor impulsivity showed greater reward rate and post-loss reaction-time modulation. Conclusion: Pediatric BPD is characterized by waiting impulsivity (anticipatory saccades) and elevated saccadic response variability during competitive decision-making, with impulsivity and emotional dysregulation contributing to choice variability.
Scott et al., 2017 [14]	Study type: Longitudinal observational psychophysiology study Framework used: Newcastle-Ottawa logic Key strengths: Prospective design. Ecologically valid interpersonal stress paradigm. Objective physiological measurement. Repeated symptom assessment. Key limitations: Small sample. Female-only developmental cohort. No structured BPD diagnostic assessment. Potential unmeasured family confounders. Risk of bias or quality concerns. Attrition bias unclear. Residual confounding likely. Overall quality judgment: Moderate (Fair)	Small sample. Female-only. No diagnostic interview confirmation	NIMH grants (K01 MH086713; R01 MH101088; R01 MH056630; K01 MH101289; K01 MH086811; F32 MH102895). NIDA (R01 DA012237). Office of Juvenile Justice and Delinquency Prevention (2013-JF-FX-0058). FISA Foundation. Falk Fund. No conflicts declared.	Ethics approval: NR Informed consent: NR Vulnerable populations: Adolescents included Risk–benefit balance: Minimal psychological stress exposure Privacy/data protection: NR Compensation: NR Trial registration: NR Protocol deviations: NR Data availability: NR Preregistration: NR Selective reporting risk: Possible Generative AI use: NR Overall ethical transparency judgment: Unclear	Greater pupillary response to criticism predicted increases in BPD symptoms over time. Greater pupillary and positive affective response to praise associated with higher baseline symptoms but faster symptom decline (context-dependent effects) Conclusion: Physiological reactivity to interpersonal feedback may function as both risk and protective factor in BPD symptom development depending on context
Seitz et al., 2021 [6]	Study type: Cross-sectional experimental cognitive study Framework used: Newcastle-Ottawa logic Key strengths: Unmedicated patient sample. Matched controls. Objective eye-tracking outcomes. Validated trauma assessment. Key limitations: Female-only. No psychiatric control group. Subsample analyses reduce power. Static facial stimuli. Risk of bias or quality concerns. Residual confounding from comorbid anxiety disorders. Possible selection bias. Overall quality judgment: Moderate (Fair-Good)	Female-only. Comorbidity high. No clinical control group. Subsample analyses for saccade latency	German Research Foundation grants	Ethics approval: Yes (Heidelberg Ethics Committee) Informed consent: Written Vulnerable populations: Psychiatric patients Risk–benefit balance: Minimal Privacy/data protection: NR Compensation: Reimbursement reported Trial registration: NR Protocol deviations: Equipment-related exclusions Data availability: On request Preregistration: NR Selective reporting risk: Possible Generative AI use: NR Overall ethical transparency judgment: Adequate	No overall group difference in emotion recognition accuracy (~93%). BPD showed more initial saccades in brief condition (F=6.62 p=.012 η^2=.09). Faster saccades toward eyes (F=8.16 p=.008 η^2=.24). No longer fixation duration on eyes vs HC. Conclusion: Findings suggest generalized visual hypervigilance to social cues and ACE-related anger bias in BPD
Wenk et al., 2025 [11]	Study type: Cross-sectional laboratory experimental eye-tracking Framework used: Newcastle-Ottawa logic Key strengths: Masked priming	Comorbidities allowed. Medication heterogeneity. Female-predominant sample	No external funding (Open Access funding via Projekt DEAL). No conflicts declared	Ethics approval: NR Informed consent: Written Vulnerable populations: Psychiatric patients Risk–benefit balance: Minimal Privacy/data	No group differences in gaze parameters. Significant negative evaluation bias in BPD (group effect F(1,59)=4.98 p=0.029 η_p^2=0.08). Early dwell time priming effect across groups (prime×AOI F≈4.33 p=0.004

Study	Quality appraisal of the study	Risk of bias / quality notes	Funding / conflicts of interest	Ethical considerations	Main findings and conclusion
	paradigm (automatic processing focus). Matched control group. Objective attention measures. Rigorous diagnostic interviews. Key limitations: Moderate sample size. Comorbid disorders and medication. Cross-sectional design. Laboratory ecological validity. Risk of bias or quality concerns. Residual confounding. Selection bias possible. Overall quality judgment: Moderate (Fair-Good)			protection: NR Compensation: Financial reimbursement reported Trial registration: NR Protocol deviations: Exclusions for prime awareness and calibration Data availability: NR Preregistration: NR Selective reporting risk: Moderate Generative AI use: NR Overall ethical transparency judgment: Limited	ηp^2=0.07) Conclusion: BPD characterized by general negative interpretation bias but not altered automatic gaze orienting to masked emotional stimuli
Wenk et al., 2024 [10]	Study type: Cross-sectional laboratory eye-tracking Framework used: Newcastle-Ottawa logic Key strengths: Large sample for eye-tracking BPD research. Matched control group. Free-viewing paradigm capturing endogenous attention. Validated diagnostic interviews. Key limitations: High comorbidity burden. Medication exposure. No psychiatric control group. Early attention parameter reliability limitations. Risk of bias or quality concerns. Residual confounding. Possible selection bias. Overall quality judgment: Moderate (Fair-Good)	High psychiatric comorbidity. Medication heterogeneity. Free-viewing paradigm limits avoidance interpretation	No external funding	Ethics approval: Yes (Leipzig Medical School ethics committee DE/EKSN40) Informed consent: Written Vulnerable populations: Psychiatric patients Risk–benefit balance: Minimal Privacy/data protection: NR Compensation: Financial reimbursement Clinical trial registration: NR Protocol deviations: COVID-related exclusions Data availability: On request Preregistration: NR Selective reporting risk: Moderate Generative AI use: NR Overall ethical transparency judgment: Adequate	Shorter dwell time on happy faces in BPD (t(84)=2.66 p=.005 d=0.57). No group differences in dwell time for angry. sad. neutral faces. No group differences in entry times for any expression. Entry time shortest for angry faces across groups (threat vigilance effect F(3,252)=21.48 p<.001 ηp^2=.20). Conclusion: BPD characterized by reduced sustained attention to positive facial expressions. No evidence for early threat vigilance

Note. NR = not reported. The appraisal framework applied to each study (RoB 2 for randomised controlled trials; Newcastle–Ottawa Scale logic for observational case-control and cohort studies) is stated in the “Quality appraisal of the study” column. Overall quality corresponds to the harmonised four-level scale (Low, Moderate, Some concerns, High) defined in Section 2 of the main text. Reference numbers in square brackets match the reference list of the main manuscript.
